# Elevated miR‐124‐3p in the aging colon disrupts mucus barrier and increases susceptibility to colitis by targeting T‐synthase

**DOI:** 10.1111/acel.13252

**Published:** 2020-10-11

**Authors:** Li Huang, Ting‐yi Sun, Liang‐jun Hu, Shi‐long Hu, Hai‐mei Sun, Fu‐qian Zhao, Bo Wu, Shu Yang, Feng‐qing Ji, De‐shan Zhou

**Affiliations:** ^1^ Department of Histology and Embryology, School of Basic Medical Sciences Capital Medical University Beijing China; ^2^ Beijing Key Laboratory of Cancer Invasion and Metastasis Research Beijing China; ^3^ Cancer Institute of Capital Medical University Beijing China

**Keywords:** aging, colitis, glycosyltransferase, microRNA, mucus

## Abstract

The risk of colitis and colorectal cancer increases markedly throughout adult life, endangering the health and lives of elderly individuals. Previous studies have proposed that bacterial translocation and infection are the main risk factors for these diseases. Therefore, in the present study, we aimed to identify the underlying mechanism by focusing on the mucus barrier function and mucin‐type *O*‐glycosylation. We evaluated alterations in the colon mucus layer in 2‐, 16‐, and 24‐month‐old mice and aged humans. Aged colons showed defective intestinal mucosal barrier and changed mucus properties. The *miR*‐*124*‐*3p* expression level was significantly increased in the aged distal colonic mucosa, which was accompanied by an increase in pathogens and bacterial translocation. Meanwhile, T‐synthase, the rate‐limiting enzyme in *O*‐glycosylation, displayed an age‐related decline in protein expression. Further experiments indicated that *miR*‐*124*‐*3p* modulated *O*‐glycosylation by directly targeting T‐synthase. Moreover, young mice overexpressing *miR*‐*124*‐*3p* exhibited abnormal glycosylation, early‐onset, and more severe colitis. These data suggest that *miR*‐*124*‐*3p* predisposes to senile colitis by reducing T‐synthase, and the *miR*‐*124*‐*3p*/T‐synthase/*O*‐glycans axis plays an essential role in maintaining the physiochemical properties of colonic mucus and intestinal homeostasis.

## Graphical abstract

Young colons show intact inner layer and mucin‐type *O*‐glycosylation, limiting microbiota access to the epithelium. Age‐related alteration in *miR‐124‐3p*/T‐synthase/*O*‐glycans axis and reduction in mucus thickness weakened the mucus barrier, leading to abnormal bacterial intrusion and increased susceptibility to senile colitis.

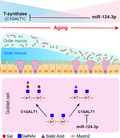

## 1 INTRODUCTION

According to the United Nations 2019 Revision of World Population Prospects, the proportion of people over 65 years of age is expected to increase from approximately 9% in 2019 to nearly 16% in 2050; thus, global population aging is the greatest challenge for further global public health efforts in the future. Aging is a natural process characterized by progressive functional impairment of tissues and organs and even pathophysiological changes. In particular, the gastrointestinal (GI) tract is greatly impacted by aging. A large amount of clinical research has indicated that age‐mediated degenerative alterations in the physiological function of the gut can cause a variety of GI diseases, such as polyps, inflammatory bowel disease (IBD), and tumors. Of these diseases, IBD, including ulcerative colitis (UC) and Crohn's disease (CD), has evolved into a global disease, with rising incidence and prevalence on every continent (Ashwin et al., 2020; Chi, 2016; Kaplan, 2015). As the global population ages, the number of older people living with IBD increases markedly, and these individuals often suffer from a high risk of developing colorectal cancer (Ha & Katz, 2014). The precise etiology of IBD remains elusive, but gut dysfunction is strongly correlated with the pathogenesis of IBD. Our recent work (Sun et al., 2018) demonstrated that the number of interstitial cells of Cajal and enteric neurons in the entire digestive tract is reduced with increasing age, which might account for GI motility malfunction and attenuated digestion and absorption functions. Further experiments indicated that the age‐related changes could be due to the inflammatory state in their microenvironment (Sun et al., 2018), suggesting that aging is correlated with the genesis and development of IBD and associated tumors. Mounting evidence has shown that the breakdown of intestinal homeostasis, which depends on the intestinal mucosal barrier and dynamic crosstalk among the intestinal microbiota, intestinal epithelial cells, and the host immune system, majorly contributes to the pathogenesis of inflammation (Maloy & Powrie, 2011; Sartor & Wu, 2017; Shin & Kim, 2018); however, the underlying mechanism is still not fully clarified.

The intestinal mucosal barrier integrity is essential for maintaining normal intestinal homeostasis. Physiologically, the thick mucus covering the epithelium, which is synthesized by goblet cells, is placed at the center of interactions between the epithelial immune system and luminal pathogens and is the primary defensive layer separating microbiota from epithelial cells. In a healthy colon, mucus comprises two layers; the dense inner layer is virtually free of bacteria and prevents bacteria from penetrating due to acting as a size‐exclusion filter, while the loose outer layer is heavily colonized by the gut microbiota (Johansson & Hansson, 2016). Gel‐forming mucin2 (MUC2), which is the major building block of mucus, is a large glycoprotein characterized by abundant and variable *O*‐linked oligosaccharides (*O*‐glycans), which account for up to 80% of the mass of mucin molecules and are responsible for the gel‐like properties of mucus (Fu et al., 2011; Johansson et al., 2008). Recent studies have revealed that the biosynthesis of mucin‐type *O*‐glycans is initiated by polypeptide α‐N‐acetylgalactosaminyltransferase (ppGalNAcT), which transfers GalNAc to specific serine or threonine (Ser/Thr) residues to generate intermediate Thomsen‐nouvelle (Tn) antigen (GalNAcα1‐Ser/Thr), which is then normally extended to form the main type of *O*‐glycans, core 1‐derived structures (also called Thomsen‐Friedenreich antigen or T antigen). T‐synthase (core 1 *β*1,3‐galactosyltransferase) is the key enzyme in the process of core 1‐derived glycans, and the active form of T‐synthase requires the co‐expression of the unique molecular chaperone Cosmc (Fu et al., 2011; Ju & Cummings, 2002). Therefore, factors regulating the expression of T‐synthase and Cosmc are of interest because they can lead to an inability to synthesize core‐1 *O*‐glycan and the deposition of Tn antigens, which potentially contribute to several autoimmune diseases, including IgA nephropathy and Tn syndrome (Berger, 1999; Serino et al., 2012). The Tn antigen is also recognized as a tumor‐associated antigen in pancreatic cancer and colitis‐associated colon cancer (Bergstrom et al., 2016; Chugh et al., 2018; Hofmann et al., 2015). However, it has remained unclear whether the aberrant glycosylation of mucins is associated with the increased susceptibility of elderly individuals to microbiota dysbiosis and IBD compared to that in younger individuals.

Since the discovery of microRNAs (miRNAs), extensive research has shown that miRNA molecules are important regulators of gene expression as a part of the epigenetic machinery (Moutinho & Esteller, 2017). Clinically, individual miRNAs are dysregulated in almost all diseases, and much attention has been paid to the role of miRNAs in colitis and inflammation‐related gut cancers (Tili et al., 2017). To date, great effort has focused on deciphering functions of miRNAs on validated target transcripts in the gut immune system (regulatory T cells, macrophages, inflammasomes, etc.) and the epithelial barrier (Heinsbroek et al., 2016; Neudecker et al., 2017; Wang et al., 2016; Yang et al., 2016). Nevertheless, the miRNAs implicated in regulating the properties of intestinal mucus and bacterial invasion remain obscure.

Here, to explore the relationships among miRNAs, colonic mucus properties and intestinal homeostasis during aging, we performed studies in both naturally aging mice and human tissues. We focused on determining the mucus properties in aging and the interaction between *miR*‐*124*‐*3p* and T‐synthase gene. To further assess the role of *miR*‐*124*‐*3p*, we used *miR*‐*124*‐*3p* agomir‐treated mice in conjunction with oral dextran sulfate sodium (DSS) administration to model colitis.

## 2 RESULTS

### 2.1 Aged colon exhibits a defective mucosal barrier

**Figure 1:**
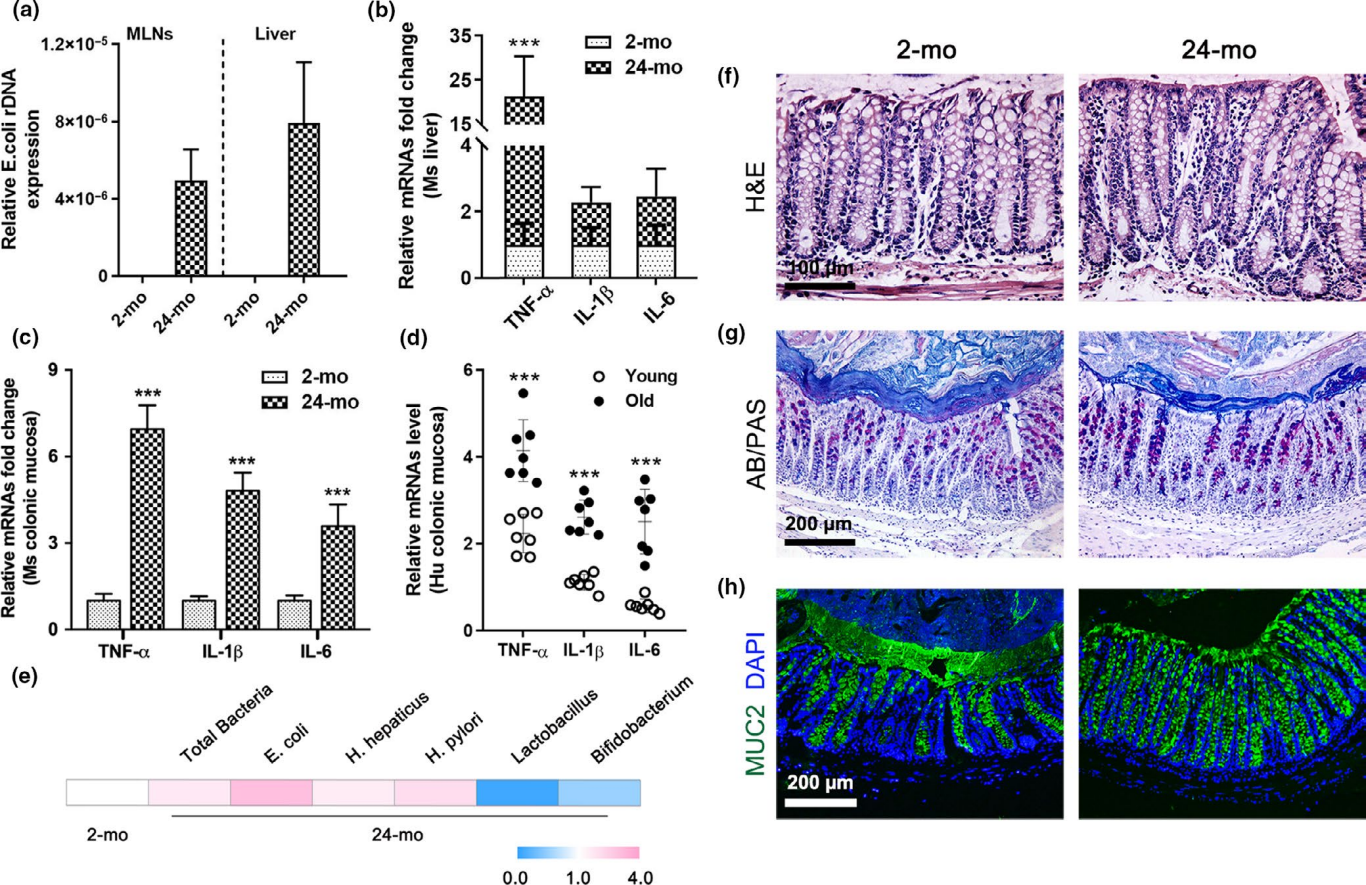
Aged colons exhibit microbiota dysbiosis and a thinner mucus layer. (a) Real‐time PCR analysis of pathogenic *E*.* coli* detected in the MLNs and liver of 24‐mo‐old mice but not 2‐mo‐old mice. (b–d) Relative mRNA fold changes or levels of TNF‐α, IL‐1β, and IL‐6 in young and old mouse livers (b) and the distal colonic mucosa of mice (c) and humans (d). (e) Relative fold changes of common bacteria in the distal colonic mucosa in old mice compared to young mice (standardized to 18S rRNA). (f) Representative H&E staining images of the distal colon from 2‐ and 24‐mo‐old mice. (g, h) Images showing AB/PAS staining (g) and MUC2 immunofluorescence staining (h) in the distal colons of young and old mice. Data are presented as mean ± SD. ***p < 0.001 compared with 2‐mo‐old mouse group or young people group. Student's *t* test or Mann–Whitney *U* test for (b–d). *n* = 8 mice or 7 human samples per group. Abbreviations: Ms, mouse; Hu, human.

We first evaluated the intestinal mucosal barrier function during aging. At 24 months (mo) of age, we found marked translocation of *E*.* coli* inhabiting the mucus layer to the mesenteric lymph nodes (MLNs) and liver (Figure 1a), accompanied by increased proinflammatory cytokines, including tumor necrosis factor‐α (TNF‐α), interleukin (IL)‐1β, and IL‐6, in the colonic mucosa in both old mice and elderly population, we also observed increases in TNF‐α in the mouse liver (Figure 1b–d). Moreover, aging of the distal colonic mucosa led to a prominent increase in the numbers of adherent total bacteria based on bacteria analysis, and the numbers of enteropathogenic bacteria *E*.* coli*, *Helicobacter hepaticus* (*H*.* hepaticus*), and *Helicobacter pylori* (*H*.* pylori*) increased, while the levels of genus *Lactobacillus* and *Bifidobacterium* as probiotics decreased in the elderly (Figure 1e). Nevertheless, the epithelial microstructure was intact, and there was no epithelial damage or ulceration associated with aging (Figure 1f). However, the mucus thickness was greatly reduced in old mice as shown by Alcian blue/periodic acid‐Schiff (AB/PAS) staining (Figure 1g, Figure S1A) and MUC2 immunofluorescence assay (Figure 1h, Figure S1B), but the number of goblet cells in the colon was unchanged with increasing age (Figure S1C).

### 2.2 Aged distal colon epithelium exhibits low levels of T‐synthase and a breached inner mucus gel layer

**Figure 2:**
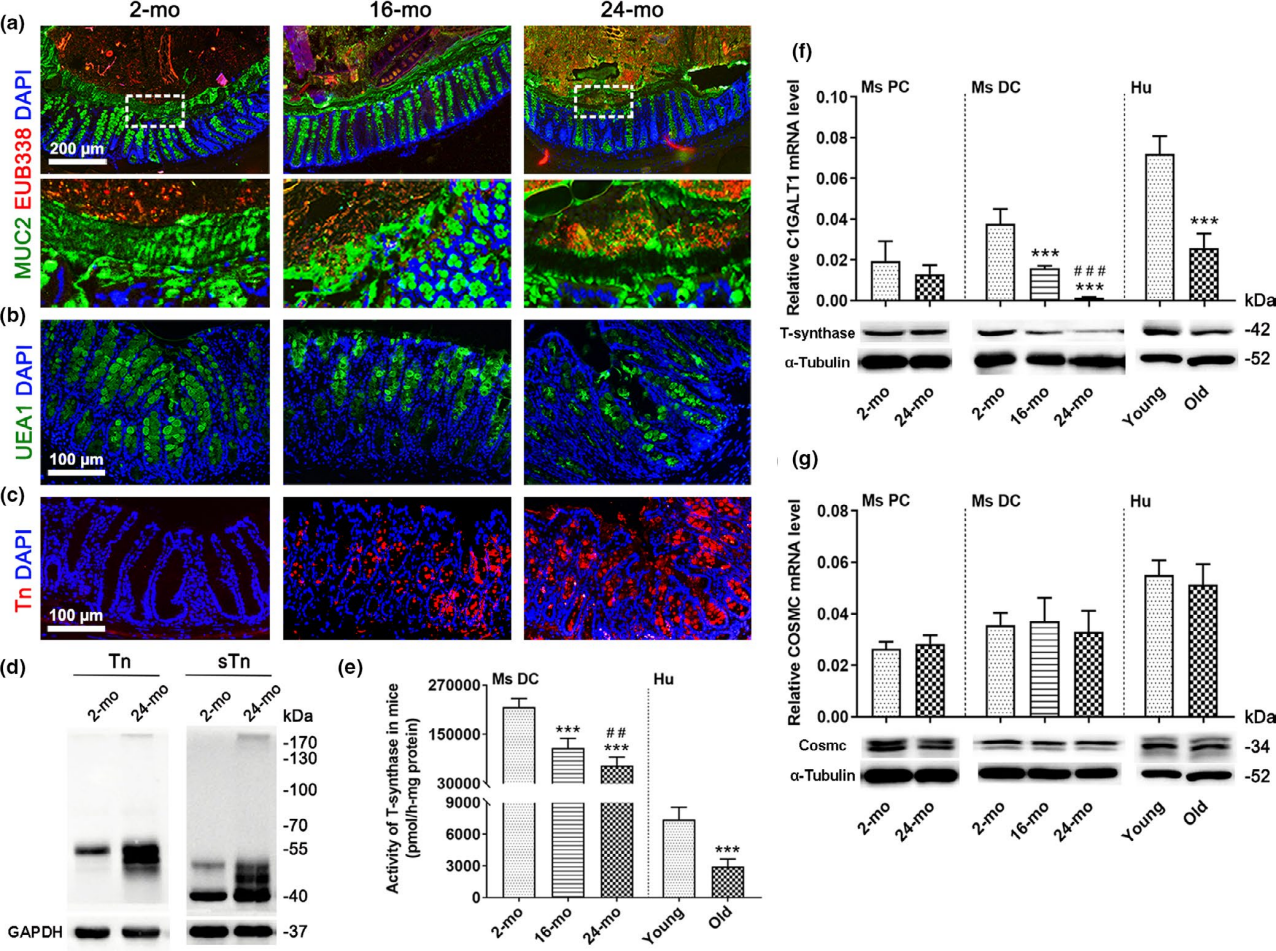
Aged distal colon shows impaired mucus barrier integrity and low level of T‐synthase. (a) FISH using EUB338 probe and MUC2 antibody on distal colonic sections of mice showing bacterial penetration into the inner mucus layer. The magnified images in the 2‐ and 24‐mo‐old mouse groups showing boxed regions. The magnified image in the 16‐mo‐old mouse group revealing bacterial penetration of the only 16‐mo‐old mouse. (b) Representative fluorescent images showing UEA1 staining in the distal colons of 2‐, 16‐, and 24‐mo‐old mice. (c) Immunofluorescence staining of 2‐, 16‐, and 24‐mo‐old mouse colon sections with anti‐Tn. (d) Western blot analysis showing the high expression of Tn antigen and sTn antigen in the old mouse distal mucosa compared to young. (e) T‐synthase activity in the mouse distal colon and human colon. (f, g) Relative mRNA expression of *C1GALT1* (f) and *COSMC* (g) and Western blot analysis of T‐synthase (f) and Cosmc (g) protein expression in the mouse and human colon. Data are presented as mean ± SD. *** *p* < 0.001 compared with 2‐mo‐old mouse group or young people group; ## *p* < 0.01, ### *p* < 0.001 compared with 16‐mo‐old mouse group. Student's *t* test for (e‐g Ms PC and Hu), and ANOVA and LSD test for (e‐g Ms DC). *n* = 8 mice or 7 human samples per group. Abbreviations: Ms, mouse; Hu, human; PC, proximal colon; DC, distal colon.

The bacterial translocation in the aged distal colon was evaluated by fluorescence *in situ* hybridization (FISH). Surprisingly, the aged (24 month) colonic mucus layer allowed bacteria to penetrate. One of the 16‐month‐old mice also showed a defective inner layer, but the inner mucus layer was intact and sterile in all 2‐month‐old mice and the other four 16‐mo‐old mice (Figure 2a). It is known that fucose is a common capping structure on *O*‐glycans, and immature *O*‐glycans truncated to Tn antigen does not carry any fucose; thus, we used lectin *Ulex europaeus* agglutinin‐1 (UEA1, binds Fucα1,2 Gal epitopes) staining to detect *O*‐glycan structures. The reduction in UEA1 in goblet cells that occurred with aging is shown in Figure 2b and Figure S2A, suggesting the loss of *O*‐glycans. The Tn antigen is a frequently occurring truncated immature *O*‐glycan that expressed at high levels in many diseases. As expected, the specific expression of the Tn antigen was observed in intracellular space in aged mice, and it was barely detectable in young tissues (Figure 2c, Figure S2B). The Western blot analysis of the Tn antigen and its sialylated form, sialyl‐Tn (sTn) antigen, also showed increased expression during aging (Figure 2d, Figure S2C). As a critical factor of subsequent *O*‐glycosylation, T‐synthase activity in the distal colonic mucosa was notably decreased in both aged mice and elderly individuals (Figure 2e). Because T‐synthase and its molecular chaperone Cosmc are required for the *O*‐glycosylation of MUC2, we then performed their mRNA and protein expression analyses. The levels of T‐synthase and its mRNA *C1GALT1* were markedly decreased in old mice and humans, but only in the distal colon, excluding the proximal colon (Figure 2f, Figure S2D). In contrast, neither the protein nor mRNA expression of Cosmc changed in the aged colon (Figure 2g, Figure S2E). Owing to these findings, the emphasis here is on T‐synthase.

### 2.3 *MiR*‐*124*‐*3p* is a direct regulator of the *C1GALT1* and downregulates T‐synthase expression

**Figure 3:**
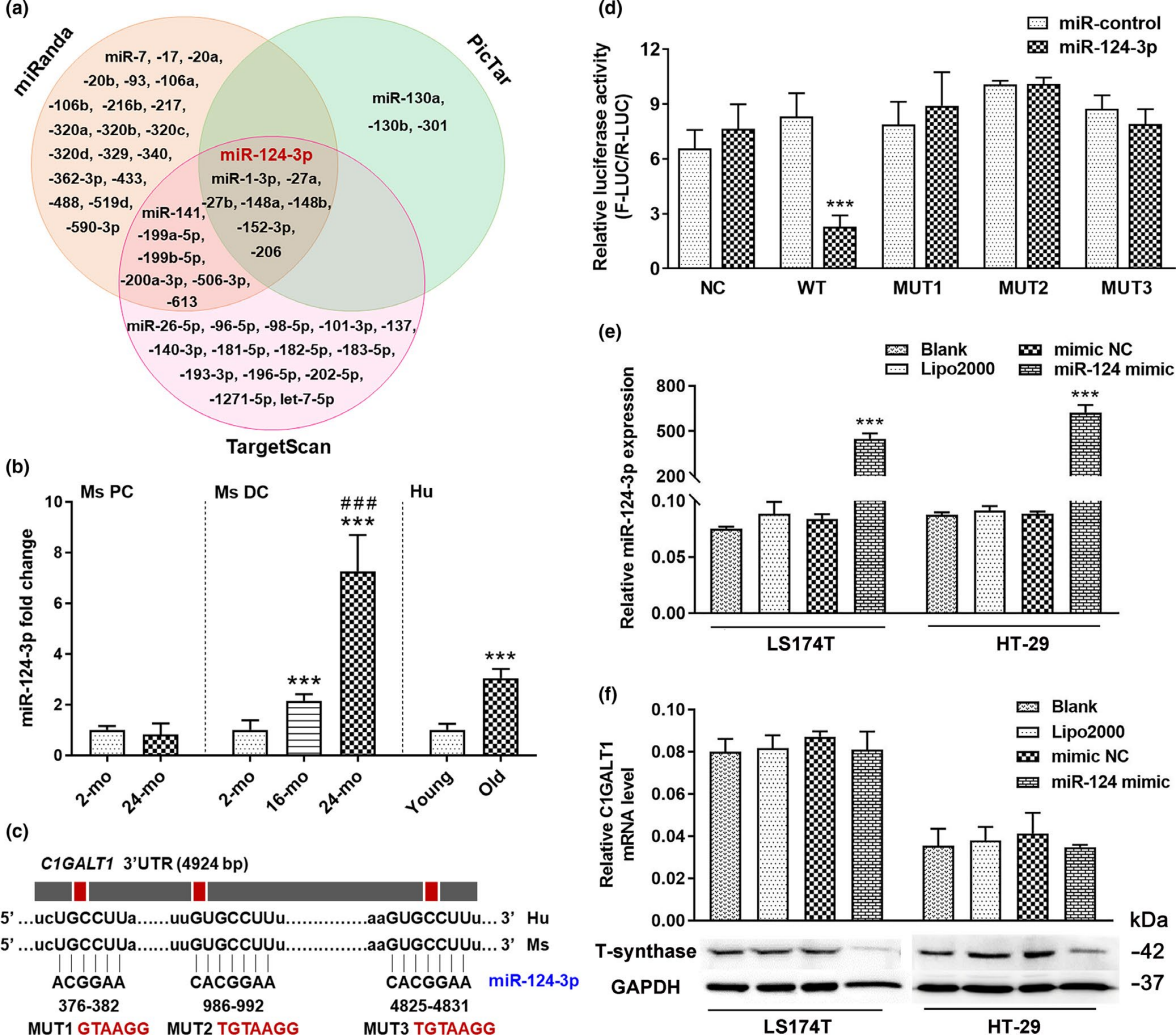
*MiR*‐*124*‐*3p* downregulates T‐synthase expression. (a) Presumable miRNAs that regulate *C1GALT1* as identified from the public databases PicTar, TargetScan, and miRanda. (b) *MiR*‐*124*‐*3p* expression was higher in old mice and aged human colon. Data are presented as mean ± SD. *** *p* < 0.001 compared with 2‐mo‐old mouse group or young people group; ### *p* < 0.001 compared with 16‐mo‐old mouse group. Student's *t* test for (Ms PC and Hu), and ANOVA and LSD test for (Ms DC). *n* = 8 mice or 7 human samples per group. (c) Putative *miR*‐*124*‐binding sites in the *C1GALT1* 3'UTR based on TargetScan analysis and mutated 3'UTR sequences of *C1GALT1*. (d) The results of the dual‐luciferase reporter assay. Data are presented as mean ± SD. *** *p* < 0.001 compared with miR‐control group (ANOVA and LSD test). *n* = 3 per group. (e) Real‐time PCR analysis of *miR*‐*124*‐*3p* in cells transfected with *miR*‐*124*‐*3p* mimic and NC. Data are presented as mean ± SD. *** *p* < 0.001 compared with mimic NC group (ANOVA and LSD test). *n* = 3 per group. (f) Relative expression of *C1GALT1*, and Western blotting showing low expression of T‐synthase protein in cells transfected with *miR*‐*124*‐*3p* mimic. Data are presented as mean ± SD (ANOVA and LSD test). *n* = 3 per group. Abbreviations: Ms, mouse; Hu, human; PC, proximal colon; DC, distal colon.

The T‐synthase is known to be directly involved in mucin‐type *O*‐glycosylation; however, the mechanism of its decreased function in aged mucosa is still unknown. In recent years, miRNAs have attracted considerable attention as regulators of gene expression (Lewis et al., 2005); therefore, we integrated the results from public databases endowed with prediction algorithms, such as PicTar (https://pictar.mdc‐berlin.de/), TargetScan (http://www.targetscan.org/vert_71/), and miRanda (http://www.microrna.org/microrna/home.do) (Figure 3a). Among 8 overlapping presumable miRNAs based on the consensus from the three algorithms, *miR*‐*124*‐*3p* caught our attention because of its high expression in senescent skin, UC, and CD (Harada et al., 2017; Qin et al., 2017; Zhao et al., 2016). Then, we found that the expression of *miR*‐*124*‐*3p* in the distal colonic mucosa was significantly increased in old mice and elderly persons, but no noticeable alterations were observed in the proximal colonic mucosa (Figure 3b), which was consistent with the results obtained from the T‐synthase test (Figure 2f, Figure S2D). Moreover, the TargetScan analysis revealed that there were three putative *miR*‐*124*‐binding sites in the *C1GALT1* 3' untranslated region (3'UTR), which are well conserved among different species (Figure 3c). To determine whether *miR*‐*124*‐*3p* is complementary to the 3'UTR sequence of *C1GALT1*, we generated luciferase reporters encoding the normal and mutated versions of the *C1GALT1* 3'UTR (Figure 3c, Figure S3A,B). Overexpression of *miR*‐*124*‐*3p* decreased the activity of the luciferase reporter encoding the 3'UTR of *C1GALT1* (Figure 3d). In contrast, the mutant *C1GALT1* 3'UTR activities were not inhibited by *miR*‐*124*‐*3p*. The relationship between *miR*‐*124*‐*3p* and *C1GALT1* was further validated in the cell line transiently transfected with *miR*‐*124*‐*3p* mimic, and the result showed a thousand‐fold increase in *miR*‐*124*‐*3p* levels compared with the mimic negative control (NC) (Figure 3e). The Western blot analysis performed on these cells showed that T‐synthase protein expression was clearly reduced (Figure 3f, Figure S3C). However, as shown in Figure 3f, the *C1GALT1* expression was unchanged, indicating that the negative regulation of T‐synthase by *miR*‐*124*‐*3p* occurred by translational suppression rather than mRNA degradation.

### 2.4 Mice overexpressing *miR*‐*124*‐*3p* show aberrant *O*‐glycans and defective mucus layer integrity

**Figure 4:**
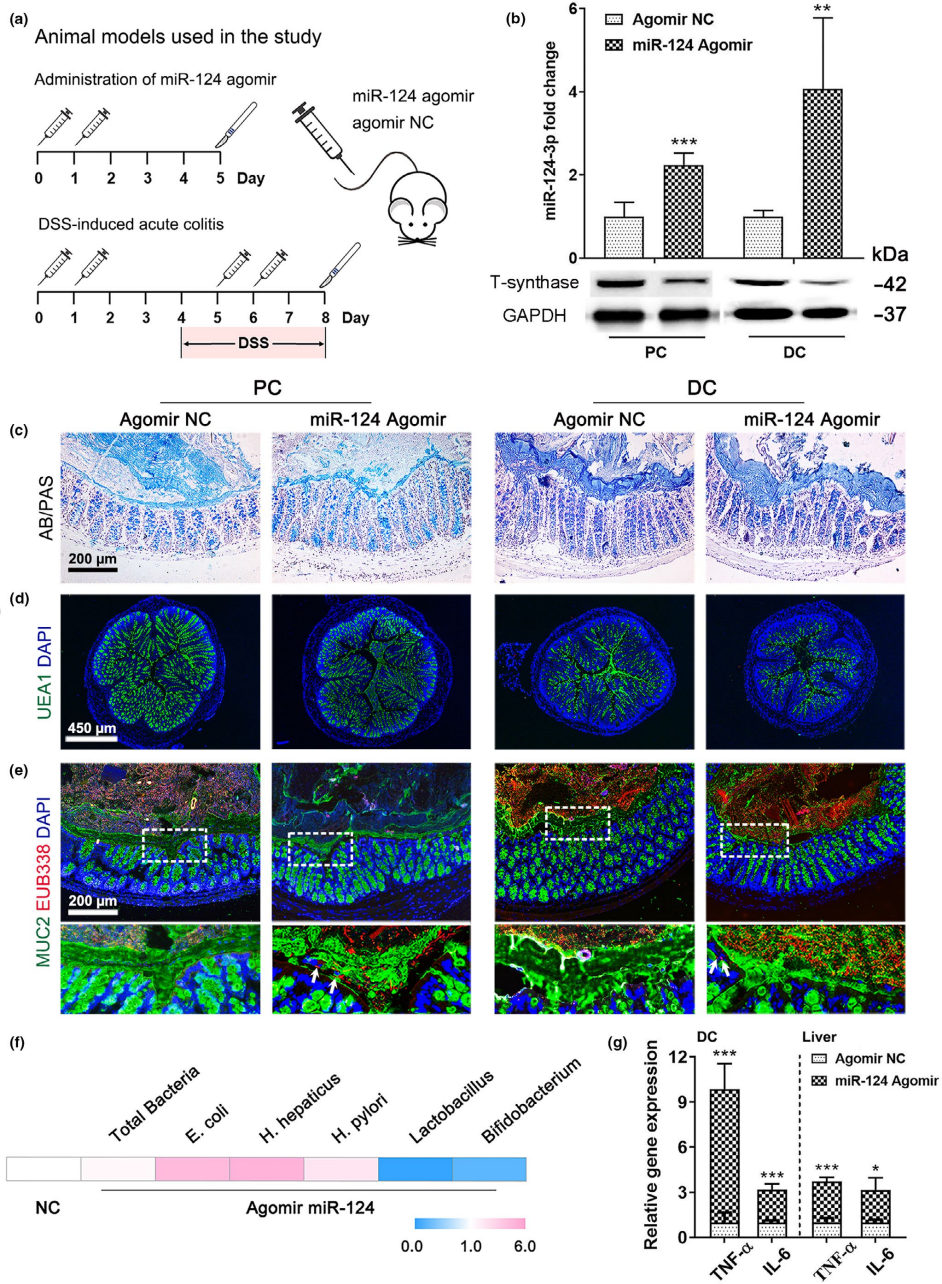
Mice overexpressing *miR*‐*124*‐*3p* show aberrant *O*‐glycans and defective integrity of the mucus layer. (a) Mouse models used in the study. (b) High expression of *miR*‐*124*‐*3p* and low T‐synthase protein expression in the proximal and distal colonic mucosa in the *miR*‐*124*‐*3p* agomir‐treated mice. (c) Representative AB/PAS staining of colonic tissues from the high *miR*‐*124*‐*3p* expression and control groups. (d) Fluorescent images showing *O*‐glycans by using UEA1 staining. (e) FISH images showing bacterial penetration into the inner mucus layer after *miR*‐*124*‐*3p* agomir administration. The magnified images below showing boxed regions. White arrows point to bacteria direct contact with the mucosal epithelium. (f) Microbiota dysbiosis in the distal colonic mucosa in *miR*‐*124*‐*3p* agomir‐treated mice. (g) High levels of TNF‐α and IL‐6 in the distal colonic mucosa and liver in the agomir‐treated group. Data are presented as mean ± SD. *p < 0.05, **p < 0.01, ***p < 0.001 compared with agomir NC group (Student's *t* test or Mann–Whitney *U* test). *n* = 6 mice per group. Abbreviations: Ms, mouse; Hu, human; PC, proximal colon; DC, distal colon.

To investigate whether the overexpression of *miR*‐*124*‐*3p* affects the properties of the intestinal mucus *in vivo*, young mice (aged 2 mo) received tail‐vein intravenous injections of *miR*‐*124*‐*3p* agomir (Figure 4a). The expression of T‐synthase was efficiently decreased both in the proximal and distal colonic mucosa in the agomir‐treated mice, while no change was found in *C1GALT1* expression (Figure 4b, Figure S4A,B). Although AB/PAS‐stained mucus layer thickness remained unaltered (Figure 4c, Figure S4C) and goblet cells had no decline in their quantity in the *miR*‐*124*‐*3p* agomir‐treated mice (Figure S4D), the UEA1 staining demonstrated that *O*‐glycans were specifically depleted after treatment with agomir (Figure 4d, Figure S4E), suggesting that the gain of *miR*‐*124*‐*3p* did not cause appreciable changes in mucus secretion but led to the loss of *O*‐glycans. We then tested whether the deficiency in *O*‐glycosylation alters the function of the mucus barrier. FISH revealed that the distal colon epithelium in the control group was covered by an intact inner layer that was devoid of bacteria. In contrast, the epithelium overexpressing *miR*‐*124*‐*3p* was in direct contact with bacteria, and the inner layer was less defined (Figure 4e). Although the proximal mucus structure was also penetrable to bacteria, it contained only a few bacteria. Moreover, compared with the control, the mice with high *miR*‐*124*‐*3p* expression exhibited microbiota dysbiosis (Figure 4f). In addition, we also observed increased levels of inflammatory gene expression (TNF‐α and IL‐6) in the distal mucosa and liver in the *miR*‐*124*‐*3p*‐overexpressing mice (Figure 4g). Collectively, our data illustrate that the leaky gut in the *miR*‐*124*‐*3p*‐overexpressing mice instigated a systemic proinflammatory response.

### 2.5 Mice overexpressing *miR*‐*124*‐*3p* show early‐onset and more severe DSS‐induced colitis

**Figure 5:**
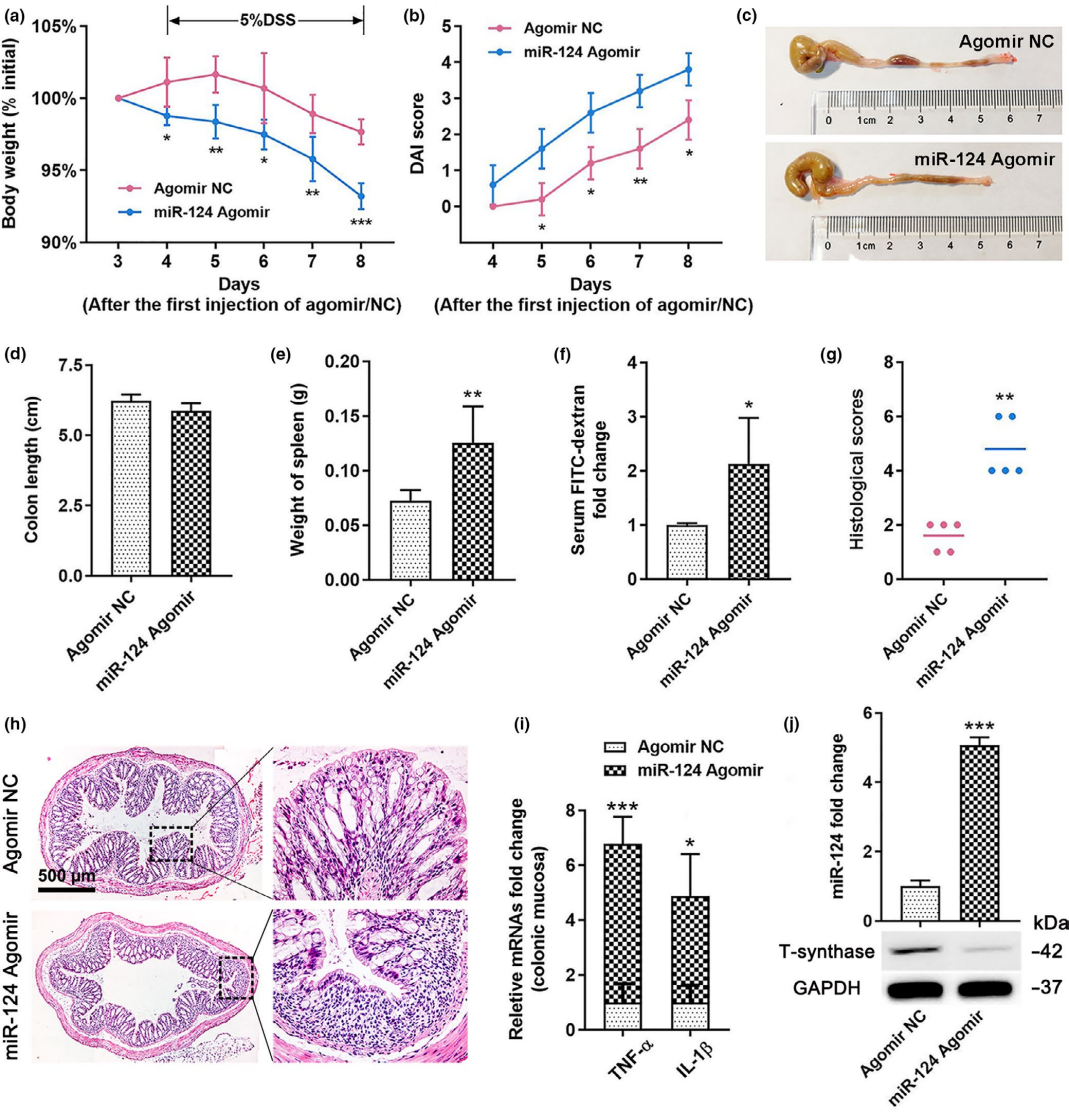
*MiR*‐*124*‐*3p* exacerbates DSS‐induced colitis. (a, b) Daily changes in body weight (a) and DAI score (b) after DSS administration. (c, d) Representative gross morphology and length analysis of the mouse colon. (e) Weight of spleen after treatment with DSS. (f) Intestinal barrier dysfunction in *miR*‐*124*‐*3p* agomir‐treated mice as detected by measuring serum FITC‐dextran. (g, h) Histologic colitis scores (g) and representative H&E images (h) of the distal colon. (i) Relative fold changes in TNF‐α and IL‐1β in the distal colonic mucosa by real‐time PCR. (j) High expression of *miR*‐*124*‐*3p* and low T‐synthase protein expression in the distal colonic mucosa of *miR*‐*124*‐*3p* agomir‐treated mice. Data are presented as mean ± SD. *p < 0.05, **p < 0.01, ***p < 0.001 compared with agomir NC group. Student's *t* test for (a,d,e,f,i,j), Mann–Whitney *U* test for (b,g). *n* = 5 mice per group.

We showed that the mucus barrier was defective in aged individuals because of *miR*‐*124*‐*3p*‐induced aberrant *O*‐glycosylation. To further determine whether increased *miR*‐*124*‐*3p* enhanced the susceptibility to colitis, an acute colitis mouse model was established by treatment with 5% DSS (Figure 4a). The mice pretreated with *miR*‐*124*‐*3p* agomir showed a tendency to lose more bodyweight and exhibited an increased disease activity index (DAI) score (Figure 5a,b), which was associated with shortening of the colon (Figure 5c,d, although it did not reach statistical significance), increased spleen weight (Figure 5e), and more permeable fluorescein isothiocyanate (FITC)‐dextran (Figure 5f), indicating worse injury. Concomitantly, compared to the control group, more severe colitis in the *miR*‐*124*‐*3p* group led to superficial inflammation in the colon, which was characterized by substantial inflammatory cellular infiltration and larger colonic mucosa collapses (Figure 5g,h, Figure S5A). Moreover, the expression of TNF‐α and IL‐1β in the agomir‐treated mice showed an increasing tendency compared to the control group (Figure 5i). Consistent with these findings, the protein level of T‐synthase was reduced, which was consistent with the high degree of *miR*‐*124*‐*3p* expression in the distal colon in the mice treated with agomir (Figure 5j, Figure S5B). These data together indicated that *miR*‐*124*‐*3p* markedly exacerbated DSS‐induced colitis.

## 3 DISCUSSION

The human gut harbors trillions of microbes, which are separated from the inner milieu of the host by the intestinal barrier. The function of this barrier mainly relies on crosstalk among the following 3 components: the commensal microbiota, the mucus layer, and the intestinal epithelium. The latter two significantly affect commensal bacterial colonization, and their defects might trigger inflammation, which is highly prevalent in elderly people. Our study showed the appearance of a gut pathobiont, *E*.*coli*, in the MLNs and liver, mirroring previous findings (Manfredo Vieira et al., 2018). These results combined with the elevated proinflammatory cytokines (TNF‐α, IL‐1β, and IL‐6) in the liver and colonic mucosa of aged mice and elderly persons suggested intestinal bacterial translocation and subsequent leakage into other visceral organs, ultimately promoting a mild inflammatory state in the elderly. However, what is the cause of the intestinal inflammation to which old people are prone? The epithelium exhibited normal morphology, and no noticeable pathologic changes in mucosal architecture were observed in old mice. The mucus layer, an important part of the intestinal barrier, was intact and robust in young mice, while bacteria were notably in direct contact with the colonic epithelium in mice aged 24 months, which was accompanied by aging‐associated gut dysbiosis, such as the shift toward proinflammatory commensals and reduction in beneficial microbes, as well as enhanced bacterial adhesion. These results illustrate that dysbacteriosis and "inflammaging" may be partly due to inadequacies in the barrier function of mucus layer, but the potential and detailed dynamic changes in gut mucus in aging people are still largely unexplored.

The impaired expression of MUC2 *O*‐glycans and deposition of Tn/sTn antigen have recently been reported to cause colonic mucus barrier breach and contribute to colitis and associated cancer (Bergstrom et al., 2016, 2017; Zhao et al., 2018). The present study showed that aged distal colons also exhibited blockade of *O*‐glycosylation, the high level of Tn/sTn antigen, and leakiness of the inner mucus. It is well known that normal *O*‐glycosylation of MUC2 relies on both the critical rate‐limiting enzyme T‐synthase and its molecular chaperone Cosmc (Fu et al., 2011; Ju & Cummings, 2002). Although Kudelka et al. (2016) reported that Cosmc was an IBD risk factor and was implicated in regulating dysbiosis in IBD, no changes in Cosmc expression levels were observed in aged humans or mice, resulting in the speculation that Cosmc might not be associated with inactive T‐synthase during aging. Importantly, we found lower enzymatic activity and decreased protein expression of T‐synthase in the aged colon. Hence, the molecular mechanism behind the impaired functioning of T‐synthase attracted our attention.

Accumulating evidence has shown the function of miRNAs as gene regulators and their involvement in the pathogenesis of gut diseases. In the present study, we found that *miR*‐*124*‐*3p* expression was significantly higher in the aged distal colon. Using the prediction algorithms TargetScan, PicTar, and miRanda, it was predicted that *miR*‐*124*‐*3p* might be bound to the 3'‐UTR of *C1GALT1*. Accordingly, we further confirmed that *miR*‐*124*‐*3p* directly targeted *C1GALT1* and epigenetically downregulated its protein levels in both *in vivo* and *in vitro* experiments. As for the reason that *C1GALT1*expression level was also significantly decreased in the aged distal colonic mucosa, we have identified that there exist other factors downregulating T‐synthase mRNA and protein expression in the aging process (unpublished data). Intriguingly, all age‐related changes in T‐synthase and *miR*‐*124*‐*3p* were observed exclusively in the distal colon, whereas the proximal segment was essentially unaltered. It has been documented that the proximal and distal colon differ in many respects, including embryological origin, local luminal environment, excretory function, and microbial flora (Flemer et al., 2017; Neumann et al., 2014; Pekow et al., 2015). There was a distinct regional expression pattern of *miR*‐*124*‐*3p*/T‐synthase in the proximal colon versus distal colon, adding to the evidence that physiological mucus heterogeneity between the proximal and distal colon exists, and is contributed to the pathological involvement of the distal colon in age‐associated diseases. Our results highlight a possible reason for this finding: The level of T‐synthase fell sharply in the aged distal colon, where numerous microorganisms live, leading to impaired glycosylation of mucins.

Importantly, the present study also serves as an additional affirmation of the relationship between *miR*‐*124*‐*3p* and the penetrability of the colonic mucus layer. Mice accumulating *miR*‐*124*‐*3p* via tail‐vein injection of agomir exhibited markedly reduced T‐synthase expression and MUC2 *O*‐glycosylation deficiency, which resulted in mucus barrier disruption and increased microbiota‐epithelial contact in both the proximal and distal colon, hinting that *miR*‐*124*‐*3p* provided a challenge to the maintenance of mucus properties and intestinal flora. Previous studies have emphasized that *Bifidobacteria* and prebiotic fiber protect against diet‐induced microbiota‐mediated colonic mucus deterioration (Schroeder et al., 2018). We observed significantly decreased probiotics and loss of intestinal *O*‐glycans in the aged colon, indicating that reasonable prebiotic‐related diet therapy assists in improving colonic mucus properties and preventing intestinal inflammation in the elderly.

Clearly, the *miR*‐*124*‐*3p*/T‐synthase/mucin glycosylation axis is related to bacterial invasion. Notably, the *miR*‐*124*‐*3p*‐treated mice showed earlier onset of experimental colitis and exacerbated inflammation after DSS administration. Combining the high levels of TNF‐α and IL‐1β in the mucosa, our results indicate that *miR*‐*124*‐*3p* was critical for internal microenvironment stability and that its high expression resulted in susceptibility to chemical‐induced inflammation. This viewpoint is also supported by recent literature showing that *miR*‐*124* might aggravate colitis by inhibiting the aryl hydrocarbon receptor in CD (Zhao et al., 2016).

In addition to the changes in mucus properties, a thinner AB/PAS‐ and MUC2‐positive mucus layer was observed overlying the mucosal surface of aged mice, which was in line with the results reported by van Beek et al. (2016). Goblet cells are the main source of intestinal gel‐forming mucins. Given that the number of goblet cells in the colon was unchanged both in the aged mice and in the *miR*‐*124*‐*3p* agomir‐treated mice, and the latter mice showed a normal inner mucus layer in terms of thickness, we believe that *miR*‐*124*‐*3p* or T‐synthase was not responsible for the thinner mucus in the aged colons, at least not directly. Our previous study indicated that SCF/c‐KIT signaling promoted intestinal mucus secretion and development of mucinous colorectal adenocarcinoma by activating PKCδ‐MARCKS (Li et al., 2018), therefore, future studies of this mechanism underlying the reduction in mucus thickness during aging are necessary. A previous study on the thickness of the mucus gel layer focused on colitis indicated that mucus thickness alone was not a useful indicator of mucus barrier function (Johansson et al., 2014). But recent research (Ahmadi et al., 2020) demonstrated that metformin increased goblet cell mass and mucin production in the older obese mouse gut, which in turn ameliorated aging‐related leaky gut and inflammation. Whatever the case is, the thinner mucus layer together with the abnormal mucus properties would make it considerably easier for microbes, food antigens, and toxins to attach to the epithelium. The long‐term and direct contact between bacteria and the mucosal epithelium might prompt some microbial species to establish themselves as residents on the surface of the mucosa and further weaken gut function during aging.

In conclusion, in the present study, the actual linkage between *miR*‐*124*‐*3p* and intestinal homeostasis is proposed. We demonstrated that *miR*‐*124*‐*3p* expression was significantly increased along with gut bacterial translocation in the distal colon in aged mice and people. Furthermore, we identified *miR*‐*124*‐*3p* as a negative regulator of *C1GALT1* and discovered that the *miR*‐*124*‐*3p*/T‐synthase/*O*‐glycans axis plays an essential role in maintaining the physiochemical properties of colonic mucus and mucosal barrier function. Overexpression of *miR*‐*124*‐*3p* induces a similar phenotype observed in old mice and increases the susceptibility to colitis in mice because of its direct modulatory effects on T‐synthase and *O*‐glycosylation. Our study provides potential targets and sheds light on new preventions and therapeutic strategies for the management of IBD in the geriatric population.

## 4 EXPERIMENTAL PROCEDURES

### 4.1 Animals

Male C57BL/6 mice were purchased from Capital Medical University and divided into three groups according to age, 2, 16, and 24 mo old (*n*  = 8 per group), to simulate the life phases at approximately 20, 50, and 70 years of age in humans (Flurkey, 2009). An additional 22 male mice (aged 2 months) were used to develop the animal model. All mice were sacrificed by appropriate anesthesia and/or cervical dislocation prior to sample collection. The colon, liver, and MLNs were surgically and aseptically removed from the mice. All animal procedures were carried out under protocols approved by the Animal Care and Use Committee of Capital Medical University (Permit Number AEEI‐2016‐143, 17 October 2016).

### 4.2 Human tissue samples

The clinical tissue samples, including 14 normal mucosa tissue samples locating at descending colon from different colorectal cancer patients, were collected immediately after surgical resection prior to any other therapeutic intervention at Beijing Friendship Hospital, Capital Medical University (Beijing, China). All patients were chemotherapy and radiation therapy naive. The individuals in the young group were aged 27–44 years (*n* = 7, four males and three females), and the individuals in the elderly group were aged 72–81 years (*n* = 7, five males and two females). The protocol for human biospecimen collection was approved by the Clinical Research Ethics Committee of Beijing Friendship Hospital, Capital Medical University (Permit Number 2015SY12, 9 March 2015). After collection, the samples were stored at −80°C.

### 4.3 Administration of *miR*‐*124*‐*3p* agomir

Young (2‐mo‐old) mice were randomly assigned to two groups (*n* = 6 per group) and injected via the tail vein with either *miR*‐*124*‐*3p* agomir (5'‐UAAGGCACGCGGUGAAUGCC‐3', Cat.No.miR40000422‐1‐2, RiboBio, China) or agomir NC (Cat.No.miR04101‐1‐10) at a dose of 5 nmol/day for 2 consecutive days. Mice were sacrificed at 96 h after the last injection, and tissues were collected for further analyses (Figure 4a).

### 4.4 Model of DSS‐induced colitis

The procedure used to induce the DSS‐colitis model is shown in Figure 4a. Briefly, young (2‐mo‐old) mice received tail‐vein injections of *miR*‐*124*‐*3p* agomir or NC (*n* = 5 per group) daily for 2 consecutive days. This treatment was applied twice at a 4‐day interval to maintain high expression of *miR*‐*124*‐*3p*. Simultaneously, acute colitis was induced by the administration of 5% DSS (Cat.No.0216011080, MP Biomedicals, USA) in drinking water continuously for 4 days, and the mice were euthanized on day 8. The severity of colitis was assessed by body weight, disease activity index (DAI), and histological damage scores, as previously described with slight modification (Table 1). The assessment of the disease severity in the mice with DSS‐induced colitis is described in the Supplementary experimental procedures.

**Table 1:** Assessment of disease severity in DSS‐colitis mice

DAI scoring system.					
Score	Diarrheal stool score			Bloody stool score	Note
0	Normal stool			Normal colored stool	The sum of the scores of two parameters was defined as the DAI score. The scoring system ranged from 0 to 6, with 6 indicating the worst injury.
1	Mildly soft stool			Brown stool	
2	Very soft stool			Reddish stool	
3	Watery stool			Bloody stool	
Histological damage scoring system					
Parameters		Score	Histological features		Note
Inflammation		0	None		The sum of the scores of three parameters was defined as the mucosal damage score. The scoring system ranged from 0 to 10, with 10 indicating the worst injury.
		1	Slight		
		2	Moderate		
		3	Severe		
Extent of injury		0	None		
		1	Mucosa		
		2	Mucosa and submucosa		
		3	Transmural		
Crypt damage		0	None		
		1	Basal 1/3 damaged		
		2	Basal 2/3 damaged		
		3	Only surface epithelium intact		
		4	Entire crypt and epithelium lost		

### 4.5 Intestinal permeability assay

To measure gut permeability *in vivo*, mice were fasted for 4 h prior to oral FITC‐dextran (molecular mass 4 kDa, Cat.No.46944, Sigma‐Aldrich, USA) administration. Mice were gavaged with 250 mg/kg (total concentration of body weight) FITC‐dextran 4 h before sacrifice. Blood samples were collected through the tail vein, and serum was placed in a microplate reader (SpectraMax i3, Molecular Devices, USA) to determine the concentration at an excitation wavelength of 490 nm and an emission wavelength of 530 nm.

### 4.6 T‐synthase activity assay

The assessment of the T‐synthase activity was conducted using a sensitive fluorescence assay method (Ju et al., 2011). In brief, tissue extracts were prepared using homogenization buffer (Cat.No.10007063‐1, Cayman, USA) and 0.1% Triton. Then, 10 μl of tissue extracts and a total volume of 40 μl master mix containing 500 μM GalNAc‐α‐4‐(MU), 500 μM UDP‐Gal, 20 mM MnCl_2_, 0.1% Triton X‐100, 800 units of O‐glycosidase, and 50 mM MES‐NaOH buffer (pH 6.8) were added to each well of an opaque black 96‐well plate and incubated at 37°C for 1 h. Subsequently, 100 μl of 1 M Glycine‐NaOH (pH 9.6) was added to stop the reaction. The fluorescence intensity was assayed at an excitation wavelength of 355 nm and an emission wavelength of 460 nm by a SpectraMax i3 microplate reader. Reaction mix without UDP‐Gal was used as a control.

### 4.7 Detection of fucose glycoprotein

We used lectin UEA1 to monitor fucose on *O*‐glycan. Carnoy's fixed paraffin‐embedded sections were incubated with FITC‐conjugated UEA1 (FITC‐UEA1, 1:100, Cat.No.L9006, Sigma‐Aldrich, USA) at 37°C for 40 min. Then, the sections were mounted with fluorescent mounting medium containing DAPI (Cat.No.ZLI‐9557, ZSGB‐BIO, Beijing, China).

### 4.8 FISH method

For dual MUC2/FISH labeling, fecal‐pellet‐containing colon sections fixed with methanol‐Carnoy's fixative were cut (5 μm). Sections were then incubated with 30 μg cyanine3‐conjugated universal bacterial probe EUB338 (5'‐GCTGCCTCCCGTAGGAGT‐3'; bp 337‐354 in bacteria EU622773; 1 μg/μl) or a nonspecific probe (NON338, 5′‐CGACGGAGGGCATCCTCA‐3') as NC in hybridization buffer (20 mM Tris‐HCl, pH 7.4; 0.9 M NaCl; 0.1% sodium dodecyl sulfate; 20% formamide) at 50°C overnight. The sections were rinsed in wash buffer (20 mM Tris‐HCl, pH 7.4; 0.9 M NaCl) at 50°C for 20 min, followed by incubation with anti‐MUC2 (12 h at 4°C) and the secondary antibody (2 h at 4°C), as listed in Table 2.

**Table 2:** The details of antibodies used in the experiments

Antibody	Source	Dilution
For immunofluorescence staining		
MUC2	Santa Cruz, Cat.No.sc−7314	1:200
Tn antigen	GeneTex, Cat.No.GTX82968	1:50
Goat anti‐Mouse IgG (Alexa Fluor 488)	Invitrogen, Cat.No.A32723	1:400
Goat anti‐Mouse IgG (Cyanine3)	Invitrogen, Cat.No.M30010	1:300
For Western blot analysis		
T‐synthase	Abcam, Cat.No.ab180250	1:1000
Cosmc	Proteintech, Cat.No.19254‐1‐AP	1:1000
Tn antigen	GeneTex, Cat.No.GTX82968	1:500
Sialyl‐Tn (sTn)	Abcam, Cat.No.ab115957	1:300
α‐Tubulin	Proteintech, Cat.No.11224‐1‐AP	1:1000
GAPDH (for T‐synthase)	CST, Cat.No.8884	1:4000
GAPDH (for Tn and sTn)	CST, Cat.No.2118	1:2000
Goat anti‐Rabbit IgG (HRP)	Abcam, Cat.No.ab6721	1:2000
Horse Anti‐Mouse IgG (HRP)	CST, Cat.No.7076	1:2000

### 4.9 Cell culture

The human colorectal cancer cell lines, LS174T and HT‐29, as well as 293 T cell line were purchased from American Type Culture Collection (ATCC, USA). All cells were cultured in Dulbecco's modified Eagle's medium (DMEM, Cat.No.C11995500BT, Gibco, USA) supplemented with 10% fetal bovine serum (Cat.No.04‐001‐1ACS, Biological Industries, Israel) and 1% penicillin/streptomycin (Cat.No.15140122, Gibco, USA) at 37°C in the presence of 5% CO_2_.

### 4.10 Transfection of miRNA mimics

According to the manufacturer's instructions, 10 × 10^4^ LS174 T and HT‐29 cells were seeded on the 6‐well plates. Extrinsic *miR*‐*124*‐*3p* mimic (Cat.No.miR10000422‐1‐5, RiboBio, China) or the respective NC (Cat.No.miR01101‐1‐5) was transiently transfected with Lipofectamine 2000 (Cat.No.11668019, Invitrogen, USA) for 6 h and replaced with culture medium for another 72 or 96 h. Transfected cells were used for further analysis.

### 4.11 Dual‐luciferase reporter assay

The 3'‐UTR sequences of *C1GALT1* containing the predicted seed regions of *miR*‐*124*‐*3p* were chemically synthesized by GeneChem (China). The fragment was introduced into the GV306 luciferase reporter vector at the unique XbaI site, which is downstream of the *Firefly* luciferase (F‐LUC) stop codon and followed by the *Renilla* luciferase (R‐LUC) sites (Figure S3A). The seed regions of *miR*‐*124*‐*3p* in the *C1GALT1* 3’‐UTR were mutated to construct GV306‐*C1GALT1*‐MUT. The GV251‐*miR*‐*124*‐*3p* construct was created by introducing the *miR*‐*124*‐*3p* sequence into the GV251 vector at the *Bam*HI/*Hind*III sites (Figure S3B). A construct containing an unrelated miRNA was used as control and named miR‐control. The GV251‐*miR*‐*124*‐*3p* construct was transiently transfected with empty vector (NC), GV306‐*C1GALT1* (WT), or GV306‐*C1GALT1*‐MUT (MUT1, MUT2, MUT3) into 293 T cells by using Lipofectamine 2000. Forty‐eight hours after transfection following manufacturer's protocols, the cells were harvested and lysed using passive lysis buffer, and then, luciferase activity was measured using a Dual‐Luciferase Assay System (Cat.No.E1910, Promega, USA) on a SpectraMax i3 microplate reader. The ratio of F‐LUC/R‐LUC activity was calculated. The experiment was repeated three times.

### 4.12 Data analysis

Images were acquired with a fluorescence microscope (Nikon Ni, Japan) or light microscope (Leica DM LB2) and analyzed using Image‐Pro Plus 6.0 software (Media Cybernetics Inc). For the determination of mucus thickness using AB/PAS or MUC2‐stained tissue sections, 20 measurements were taken from each mouse colon with 5 mice per group. Each measurement, the vertical distance between the cell surface and the luminal mucus surface, was calculated, and the mucus thickness is clearly delineated when this layer is well preserved. The numbers of AB/PAS‐positive goblet cells were counted in 10 colonic crypts per section, and three random sections were counted per mouse, with 5 mice per group.

### 4.13 Statistical analysis

The obtained data were analyzed using one‐way analysis of variance (ANOVA) followed by LSD post hoc tests for multiple groups, and Student's *t* test (parametric data) or Mann–Whitney *U* test (nonparametric data) for two groups using SPSS 21.0 software (IBM Corporation). We analyzed the normality of data distribution and homogeneity of variance using Shapiro‐Wilk's and Levene's test, respectively. Significance was defined when the *p*‐value was < 0.05.

### 4.14 Other methods

Please see details in the Supplementary experimental procedures for assessment of disease severity in mice with DSS‐induced colitis, hematoxylin and eosin (H&E) staining, AB/PAS staining, immunofluorescence staining, Western blot analysis, and RNA and genomic DNA extraction and real‐time PCR. The primers used are listed in Table S1.

## CONFLICT OF INTEREST

The authors declare that they have no conflict of interest.

## AUTHOR CONTRIBUTIONS

T.‐y.S. and D.‐s.Z. designed the project, analyzed the data, and wrote the manuscript. L.H., T.‐y.S., L.‐j.H., S.‐l.H., and F.‐q.Z. performed the experiments. H.‐m.S., B.W., and S.Y. contributed reagents/materials/analysis tools. F.‐q.J. helped draft the manuscript.

## Supporting information

Fig S1

Fig S2

Fig S3

Fig S4

Fig S5

Table S1

Supplementary Material

Supplementary Material

## Data Availability

The data that support the findings of this study are available in the supplementary material of this article.

## References

[acel13252-bib-0001] Ahmadi, S. , Razazan, A. , Nagpal, R. , Jain, S. , Wang, B. O. , Mishra, S. P. , Wang, S. , Justice, J. , Ding, J. , McClain, D. A. , Kritchevsky, S. B. , Kitzman, D. , & Yadav, H. (2020). Metformin reduces aging‐related leaky gut and improves cognitive function by beneficially modulating gut microbiome/goblet cell/mucin axis. The Journals of Gerontology. Series A, Biological Sciences and Medical Sciences, 75(7), e9–e21. 10.1093/gerona/glaa056 PMC7302182 32129462

[acel13252-bib-0002] Ashwin, N. A. , Gilaad, G. K. , & Siew, C. N. (2020). Changing global epidemiology of inflammatory bowel diseases: sustaining health care delivery into the 21st century. Clinical Gastroenterology and Hepatology, 18(6), 1252–1260. 10.1016/j.cgh.2020.01.028 32007542

[acel13252-bib-0003] Berger, E. G. (1999). Tn‐syndrome. Biochimica Et Biophysica Acta, 1455(2–3), 255–268. 10.1016/s0925-4439(99)00069-1 10571017

[acel13252-bib-0004] Bergstrom, K. , Fu, J. , Johansson, M. E. V. , Liu, X. , Gao, N. , Wu, Q. , Song, J. , McDaniel, J. M. , McGee, S. , Chen, W. , Braun, J. , Hansson, G. C. , & Xia, L. (2017). Core 1‐ and 3‐derived O‐glycans collectively maintain the colonic mucus barrier and protect against spontaneous colitis in mice. Mucosal Immunology, 10(1), 91–103. 10.1038/mi.2016.45 27143302 PMC5097036

[acel13252-bib-0005] Bergstrom, K. , Liu, X. , Zhao, Y. , Gao, N. , Wu, Q. , Song, K. , Cui, Y. I. , Li, Y. , McDaniel, J. M. , McGee, S. , Chen, W. , Huycke, M. M. , Houchen, C. W. , Zenewicz, L. A. , West, C. M. , Chen, H. , Braun, J. , Fu, J. , & Xia, L. (2016). Defective intestinal mucin‐type O‐glycosylation causes spontaneous colitis‐associated cancer in mice. Gastroenterology, 151(1), 152–164.e11. 10.1053/j.gastro.2016.03.039 27059389 PMC5068133

[acel13252-bib-0006] Chi, K. R. (2016). Epidemiology: rising in the East. Nature, 540(7634), S100–S102. 10.1038/540S100a 28002397

[acel13252-bib-0007] Chugh, S. , Barkeer, S. , Rachagani, S. , Nimmakayala, R. K. , Perumal, N. , Pothuraju, R. , Atri, P. , Mahapatra, S. , Thapa, I. , Talmon, G. A. , Smith, L. M. , Yu, X. , Neelamegham, S. , Fu, J. , Xia, L. , Ponnusamy, M. P. , & Batra, S. K. (2018). Disruption of C1galt1 gene promotes development and metastasis of pancreatic adenocarcinomas in mice. Gastroenterology, 155(5), 1608–1624. 10.1053/j.gastro.2018.08.007 30086262 PMC6219903

[acel13252-bib-0008] Flemer, B. , Lynch, D. B. , Brown, J. M. R. , Jeffery, I. B. , Ryan, F. J. , Claesson, M. J. , O'Riordain, M. , Shanahan, F. , & O'Toole, P. W. (2017). Tumour‐associated and non‐tumour‐associated microbiota in colorectal cancer. Gut, 66(4), 633–643. 10.1136/gutjnl-2015-309595 26992426 PMC5529966

[acel13252-bib-0009] Flurkey, K. (2009). The Jackson laboratory handbook on genetically standardized mice, 6th ed The Jackson Laboratory.

[acel13252-bib-0010] Fu, J. , Wei, B. O. , Wen, T. , Johansson, M. E. V. , Liu, X. , Bradford, E. , Thomsson, K. A. , McGee, S. , Mansour, L. , Tong, M. , McDaniel, J. M. , Sferra, T. J. , Turner, J. R. , Chen, H. , Hansson, G. C. , Braun, J. , & Xia, L. (2011). Loss of intestinal core 1‐derived O‐glycans causes spontaneous colitis in mice. The Journal of Clinical Investigation, 121(4), 1657–1666. 10.1172/JCI45538 21383503 PMC3069788

[acel13252-bib-0011] Ha, C. Y. , & Katz, S. (2014). Clinical implications of ageing for the management of IBD. Nature Reviews Gastroenterology & Hepatology, 11(2), 128–138. 10.1038/nrgastro.2013.241 24345890

[acel13252-bib-0012] Harada, M. , Jinnin, M. , Wang, Z. , Hirano, A. , Tomizawa, Y. , Kira, T. , Igata, T. , Masuguchi, S. , Fukushima, S. , & Ihn, H. (2017). The expression of miR‐124 increases in aged skin to cause cell senescence and it decreases in squamous cell carcinoma. Bioscience Trends, 10(6), 454–459. 10.5582/bst.2016.01102 27818465

[acel13252-bib-0013] Heinsbroek, S. E. M. , Squadrito, M. L. , Schilderink, R. , Hilbers, F. W. , Verseijden, C. , Hofmann, M. , Helmke, A. , Boon, L. , Wildenberg, M. E. , Roelofs, J. J. T. H. , Ponsioen, C. Y. , Peters, C. P. , te Velde, A. A. , Gordon, S. , De Palma, M. , & de Jonge, W. J. (2016). miR‐511‐3p, embedded in the macrophage mannose receptor gene, contributes to intestinal inflammation. Mucosal Immunology, 9(4), 960–973. 10.1038/mi.2015.113 26530135

[acel13252-bib-0014] Hofmann, B. T. , Schlüter, L. , Lange, P. , Mercanoglu, B. , Ewald, F. , Fölster, A. , Picksak, A.‐S. , Harder, S. , El Gammal, A. T. , Grupp, K. , Güngör, C. , Drenckhan, A. , Schlüter, H. , Wagener, C. , Izbicki, J. R. , Jücker, M. , Bockhorn, M. , & Wolters‐Eisfeld, G. (2015). COSMC knockdown mediated aberrant O‐glycosylation promotes oncogenic properties in pancreatic cancer. Molecular Cancer, 14, 109 10.1186/s12943-015-0386-1 26021314 PMC4447007

[acel13252-bib-0015] Johansson, M. E. V. , Gustafsson, J. K. , Holmén‐Larsson, J. , Jabbar, K. S. , Xia, L. , Xu, H. , Ghishan, F. K. , Carvalho, F. A. , Gewirtz, A. T. , Sjövall, H. , & Hansson, G. C. (2014). Bacteria penetrate the normally impenetrable inner colon mucus layer in both murine colitis models and patients with ulcerative colitis. Gut, 63(2), 281–291. 10.1136/gutjnl-2012-303207 23426893 PMC3740207

[acel13252-bib-0016] Johansson, M. E. , & Hansson, G. C. (2016). Immunological aspects of intestinal mucus and mucins. Nature Reviews. Immunology, 16(10), 639–649. 10.1038/nri.2016.88 PMC6435297 27498766

[acel13252-bib-0017] Johansson, M. E. , Phillipson, M. , Petersson, J. , Velcich, A. , Holm, L. , & Hansson, G. C. (2008). The inner of the two Muc2 mucin‐dependent mucus layers in colon is devoid of bacteria. Proceedings of the National Academy of Sciences of the United States of America, 105(39), 15064–15069. 10.1073/pnas.0803124105 18806221 PMC2567493

[acel13252-bib-0018] Ju, T. , & Cummings, R. D. (2002). A unique molecular chaperone Cosmc required for activity of the mammalian core 1 beta 3‐galactosyltransferase. Proceedings of the National Academy of Sciences of the United States of America, 99(26), 16613–16618. 10.1073/pnas.262438199 12464682 PMC139192

[acel13252-bib-0019] Ju, T. , Xia, B. , Aryal, R. P. , Wang, W. , Wang, Y. , Ding, X. , Mi, R. , He, M. , & Cummings, R. D. (2011). A novel fluorescent assay for T‐synthase activity. Glycobiology, 21(3), 352–362. 10.1093/glycob/cwq168 20959392 PMC3033746

[acel13252-bib-0020] Kaplan, G. G. (2015). The global burden of IBD: from 2015 to 2025. Nature Reviews. Gastroenterology & Hepatology, 12(12), 720–727. 10.1038/nrgastro.2015.150 26323879

[acel13252-bib-0021] Kudelka, M. R. , Hinrichs, B. H. , Darby, T. , Moreno, C. S. , Nishio, H. , Cutler, C. E. , & Cummings, R. D. (2016). Cosmc is an X‐linked inflammatory bowel disease risk gene that spatially regulates gut microbiota and contributes to sex‐specific risk. Proceedings of the National Academy of Sciences of the United States of America, 113(51), 14787–14792. 10.1073/pnas.1612158114 27930307 PMC5187739

[acel13252-bib-0022] Lewis, B. P. , Burge, C. B. , & Bartel, D. P. (2005). Conserved seed pairing, often flanked by adenosines, indicates that thousands of human genes are microRNA targets. Cell, 120(1), 15–20. 10.1016/j.cell.2004.12.035 15652477

[acel13252-bib-0023] Li, G. , Yang, S. , Shen, P. , Wu, B. , Sun, T. , Sun, H. , & Zhou, D. (2018). SCF/c‐KIT signaling promotes mucus secretion of colonic goblet cells and development of mucinous colorectal adenocarcinoma. American Journal of Cancer Research, 8(6), 1064–1073. 30034943 PMC6048403

[acel13252-bib-0024] Maloy, K. J. , & Powrie, F. (2011). Intestinal homeostasis and its breakdown in inflammatory bowel disease. Nature, 474(7351), 298–306. 10.1038/nature10208 21677746

[acel13252-bib-0025] Manfredo Vieira, S. , Hiltensperger, M. , Kumar, V. , Zegarra‐Ruiz, D. , Dehner, C. , Khan, N. , Costa, F. R. C. , Tiniakou, E. , Greiling, T. , Ruff, W. , Barbieri, A. , Kriegel, C. , Mehta, S. S. , Knight, J. R. , Jain, D. , Goodman, A. L. , & Kriegel, M. A. (2018). Translocation of a gut pathobiont drives autoimmunity in mice and humans. Science, 359(6380), 1156–1161. 10.1126/science.aar7201 29590047 PMC5959731

[acel13252-bib-0026] Moutinho, C. , & Esteller, M. (2017). MicroRNAs and epigenetics. Advances in Cancer Research, 135, 189–220. 10.1016/bs.acr.2017.06.003 28882223

[acel13252-bib-0027] Neudecker, V. , Haneklaus, M. , Jensen, O. , Khailova, L. , Masterson, J. C. , Tye, H. , Biette, K. , Jedlicka, P. , Brodsky, K. S. , Gerich, M. E. , Mack, M. , Robertson, A. A. B. , Cooper, M. A. , Furuta, G. T. , Dinarello, C. A. , O’Neill, L. A. , Eltzschig, H. K. , Masters, S. L. , & McNamee, E. N. (2017). Myeloid‐derived miR‐223 regulates intestinal inflammation via repression of the NLRP3 inflammasome. The Journal of Experimental Medicine, 214(6), 1737–1752. 10.1084/jem.20160462 28487310 PMC5460990

[acel13252-bib-0028] Neumann, P. A. , Koch, S. , Hilgarth, R. S. , Perez‐Chanona, E. , Denning, P. , Jobin, C. , & Nusrat, A. (2014). Gut commensal bacteria and regional Wnt gene expression in the proximal versus distal colon. The American Journal of Pathology, 184(3), 592–599. 10.1016/j.ajpath.2013.11.029 24418259 PMC3936305

[acel13252-bib-0029] Pekow, J. , Meckel, K. , Dougherty, U. , Butun, F. , Mustafi, R. , Lim, J. , Crofton, C. , Chen, X. , Joseph, L. , & Bissonnette, M. (2015). Tumor suppressors miR‐143 and miR‐145 and predicted target proteins API5, ERK5, K‐RAS, and IRS‐1 are differentially expressed in proximal and distal colon. American Journal of Physiology. Gastrointestinal and Liver Physiology, 308(3), G179–G187. 10.1152/ajpgi.00208.2014 25477374 PMC4312953

[acel13252-bib-0030] Qin, Z. , Wan, J.‐J. , Sun, Y. , Wu, T. , Wang, P.‐Y. , Du, P. , Su, D.‐F. , Yang, Y. , & Liu, X. (2017). Nicotine protects against DSS colitis through regulating microRNA‐124 and STAT3. Journal of Molecular Medicine, 95(2), 221–233. 10.1007/s00109-016-1473-5 27709266

[acel13252-bib-0031] Sartor, R. B. , & Wu, G. D. (2017). Roles for Intestinal bacteria, viruses, and fungi in pathogenesis of inflammatory bowel diseases and therapeutic approaches. Gastroenterology, 152(2), 327–339.e4. 10.1053/j.gastro.2016.10.012 27769810 PMC5511756

[acel13252-bib-0032] Schroeder, B. O. , Birchenough, G. M. H. , Ståhlman, M. , Arike, L. , Johansson, M. E. V. , Hansson, G. C. , & Bäckhed, F. (2018). Bifidobacteria or fiber protects against diet‐induced microbiota‐mediated colonic mucus deterioration. Cell Host & Microbe, 23(1), 27–40.e7. 10.1016/j.chom.2017.11.004 29276171 PMC5764785

[acel13252-bib-0033] Serino, G. , Sallustio, F. , Cox, S. N. , Pesce, F. , & Schena, F. P. (2012). Abnormal miR‐148b expression promotes aberrant glycosylation of IgA1 in IgA nephropathy. Journal of the American Society of Nephrology, 23(5), 814–824. 10.1681/ASN.2011060567 22362909 PMC3338289

[acel13252-bib-0034] Shin, W. , & Kim, H. J. (2018). Intestinal barrier dysfunction orchestrates the onset of inflammatory host‐microbiome cross‐talk in a human gut inflammation‐on‐a‐chip. Proceedings of the National Academy of Sciences of the United States of America, 115(45), E10539–E10547. 10.1073/pnas.1810819115 30348765 PMC6233106

[acel13252-bib-0035] Sun, T. , Li, D. , Hu, S. , Huang, L. I. , Sun, H. , Yang, S. , Wu, B. O. , Ji, F. , & Zhou, D. (2018). Aging‐dependent decrease in the numbers of enteric neurons, interstitial cells of Cajal and expression of connexin43 in various regions of gastrointestinal tract. Aging, 10(12), 3851–3865. 10.18632/aging.101677 30530917 PMC6326649

[acel13252-bib-0036] Tili, E. , Michaille, J. J. , Piurowski, V. , Rigot, B. , & Croce, C. M. (2017). MicroRNAs in intestinal barrier function, inflammatory bowel disease and related cancers‐their effects and therapeutic potentials. Current Opinion in Pharmacology, 37, 142–150. 10.1016/j.coph.2017.10.010 29154194 PMC5938753

[acel13252-bib-0037] van Beek, A. A. , Sovran, B. , Hugenholtz, F. , Meijer, B. , Hoogerland, J. A. , Mihailova, V. , van der Ploeg, C. , Belzer, C. , Boekschoten, M. V. , Hoeijmakers, J. H. J. , Vermeij, W. P. , de Vos, P. , Wells, J. M. , Leenen, P. J. M. , Nicoletti, C. , Hendriks, R. W. , & Savelkoul, H. F. J. (2016). Supplementation with *Lactobacillus plantarum* WCFS1 prevents decline of mucus barrier in colon of accelerated aging Ercc1‐/Δ7 mice. Frontiers in Immunology, 7, 408 10.3389/fimmu.2016.00408 27774093 PMC5054004

[acel13252-bib-0038] Wang, H. , Chao, K. , Ng, S. C. , Bai, A. H. , Yu, Q. , Yu, J. , Li, M. , Cui, Y. I. , Chen, M. , Hu, J.‐F. , & Zhang, S. (2016). Pro‐inflammatory miR‐223 mediates the cross‐talk between the IL23 pathway and the intestinal barrier in inflammatory bowel disease. Genome Biology, 17, 58 10.1186/s13059-016-0901-8 27029486 PMC4815271

[acel13252-bib-0039] Yang, H.‐Y. , Barbi, J. , Wu, C.‐Y. , Zheng, Y. , Vignali, P. D. A. , Wu, X. , Tao, J.‐H. , Park, B. V. , Bandara, S. , Novack, L. , Ni, X. , Yang, X. , Chang, K.‐Y. , Wu, R.‐C. , Zhang, J. , Yang, C.‐W. , Pardoll, D. M. , Li, H. , & Pan, F. (2016). MicroRNA‐17 modulates regulatory T cell function by targeting co‐regulators of the Foxp3 transcription factor. Immunity, 45(1), 83–93. 10.1016/j.immuni.2016.06.022 27438767 PMC4957244

[acel13252-bib-0040] Zhao, M. , Xiong, X. , Ren, K. , Xu, B. , Cheng, M. , Sahu, C. , Wu, K. , Nie, Y. , Huang, Z. , Blumberg, R. S. , Han, X. , & Ruan, H.‐B. (2018). Deficiency in intestinal epithelial O‐GlcNAcylation predisposes to gut inflammation. EMBO Molecular Medicine, 10(8), pii:e8736 10.15252/emmm.201708736 PMC6079539 29941542

[acel13252-bib-0041] Zhao, Y. E. , Ma, T. , Chen, W. , Chen, Y. , Li, M. , Ren, L. , Chen, J. , Cao, R. , Feng, Y. , Zhang, H. , & Shi, R. (2016). MicroRNA‐124 promotes intestinal inflammation by targeting Aryl hydrocarbon receptor in Crohn’s disease. Journal of Crohn's & Colitis, 10(6), 703–712. 10.1093/ecco-jcc/jjw010 26802080

