# Supplementary figures and images for: Elevated miR‐124‐3p in the aging colon disrupts mucus barrier and increases susceptibility to colitis by targeting T‐synthase
Source: Aging Cell. 2020 Oct 11;19(11):e13252. doi: 10.1111/acel.13252 (PMC7681053; doi:10.1111/acel.13252)

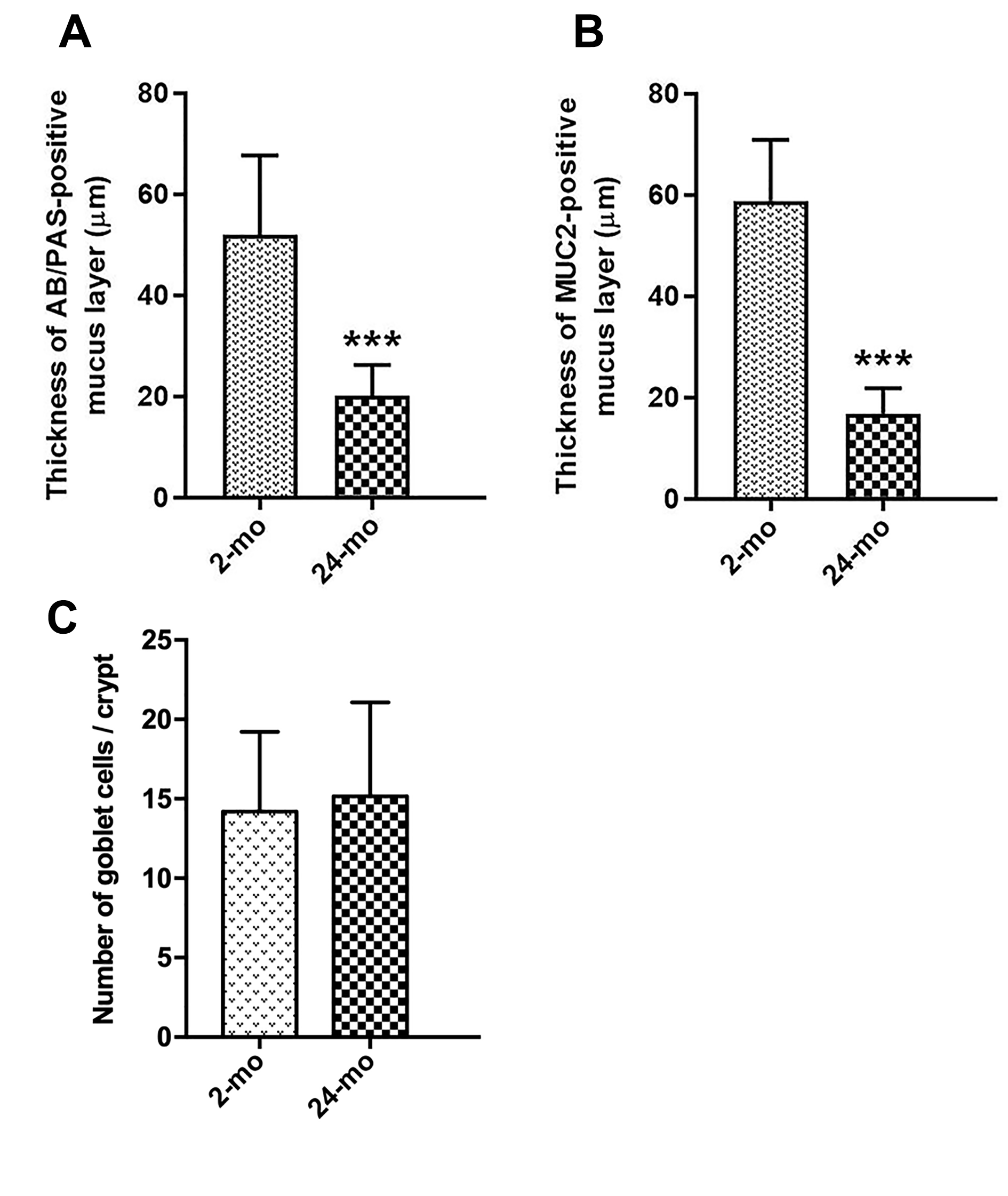

Supplement: Supplementary file 1 — Fig S1 [file ACEL-19-e13252-s001.tif]

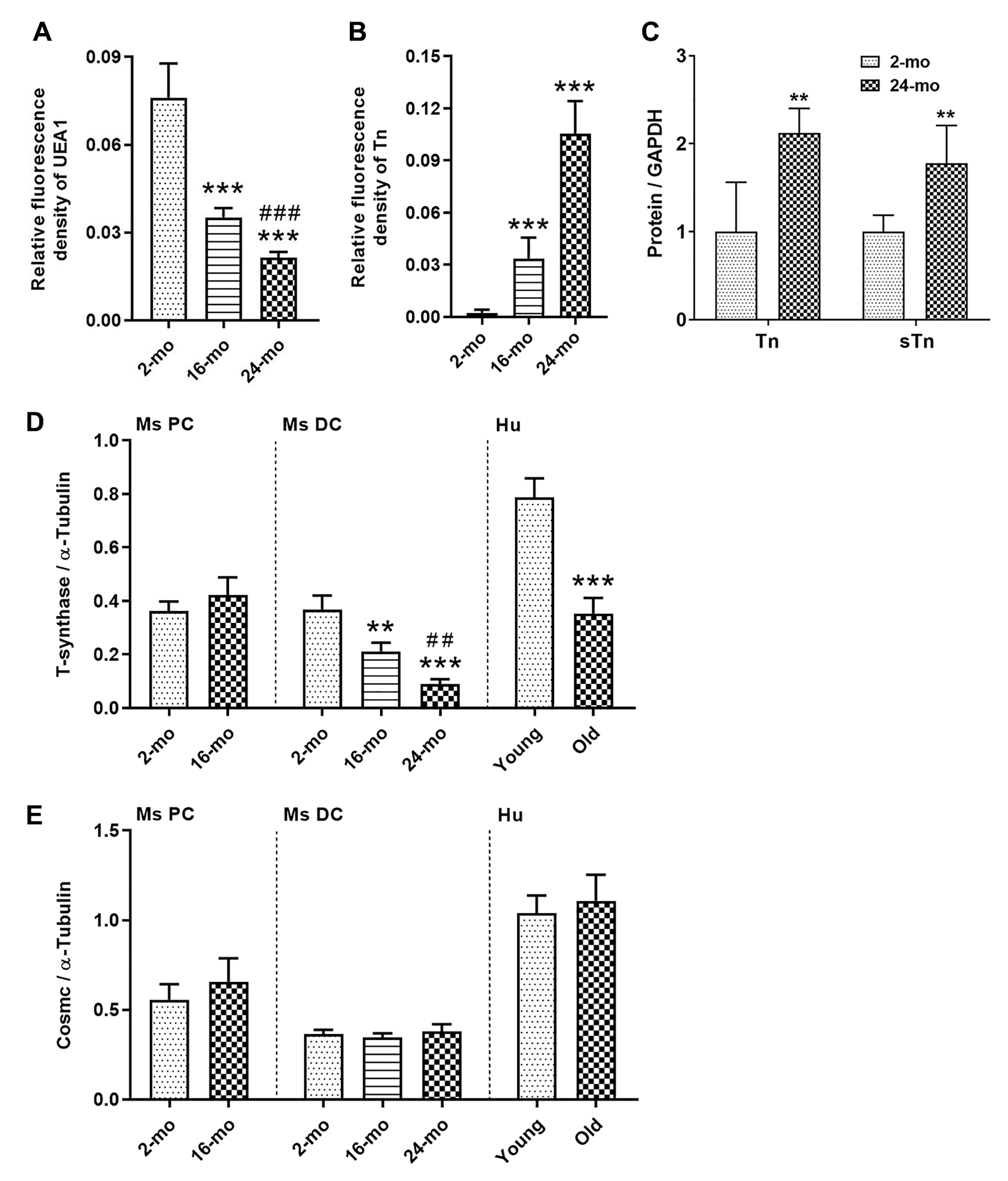

Supplement: Supplementary file 2 — Fig S2 [file ACEL-19-e13252-s002.tif]

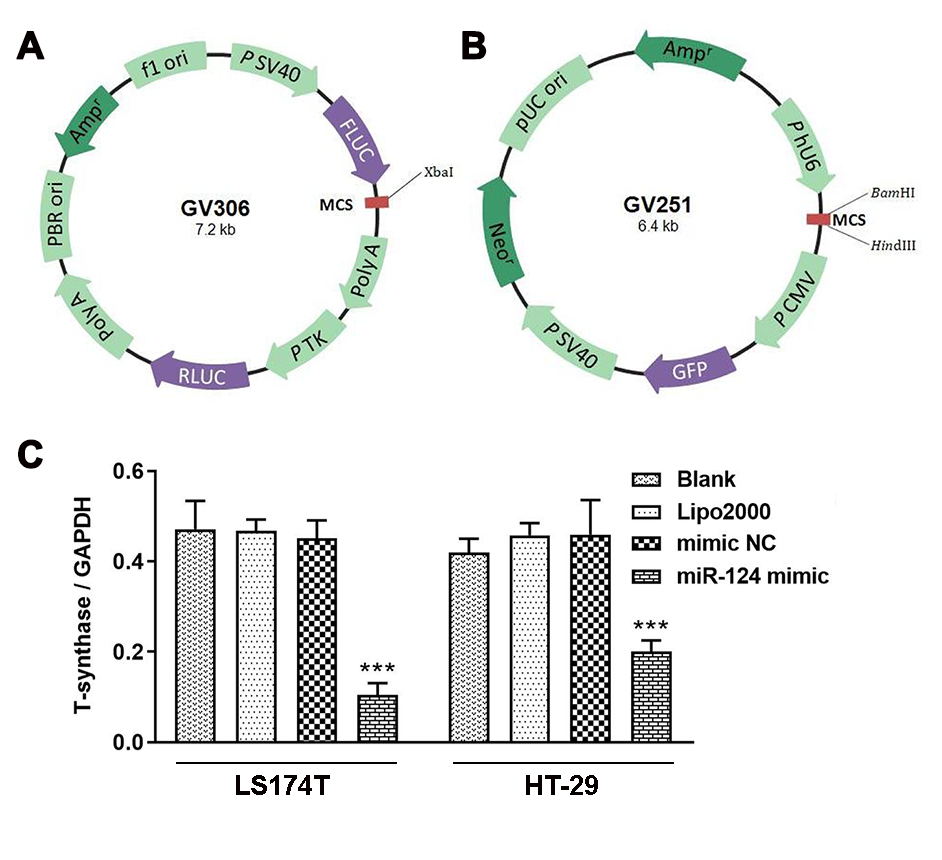

Supplement: Supplementary file 3 — Fig S3 [file ACEL-19-e13252-s003.tif]

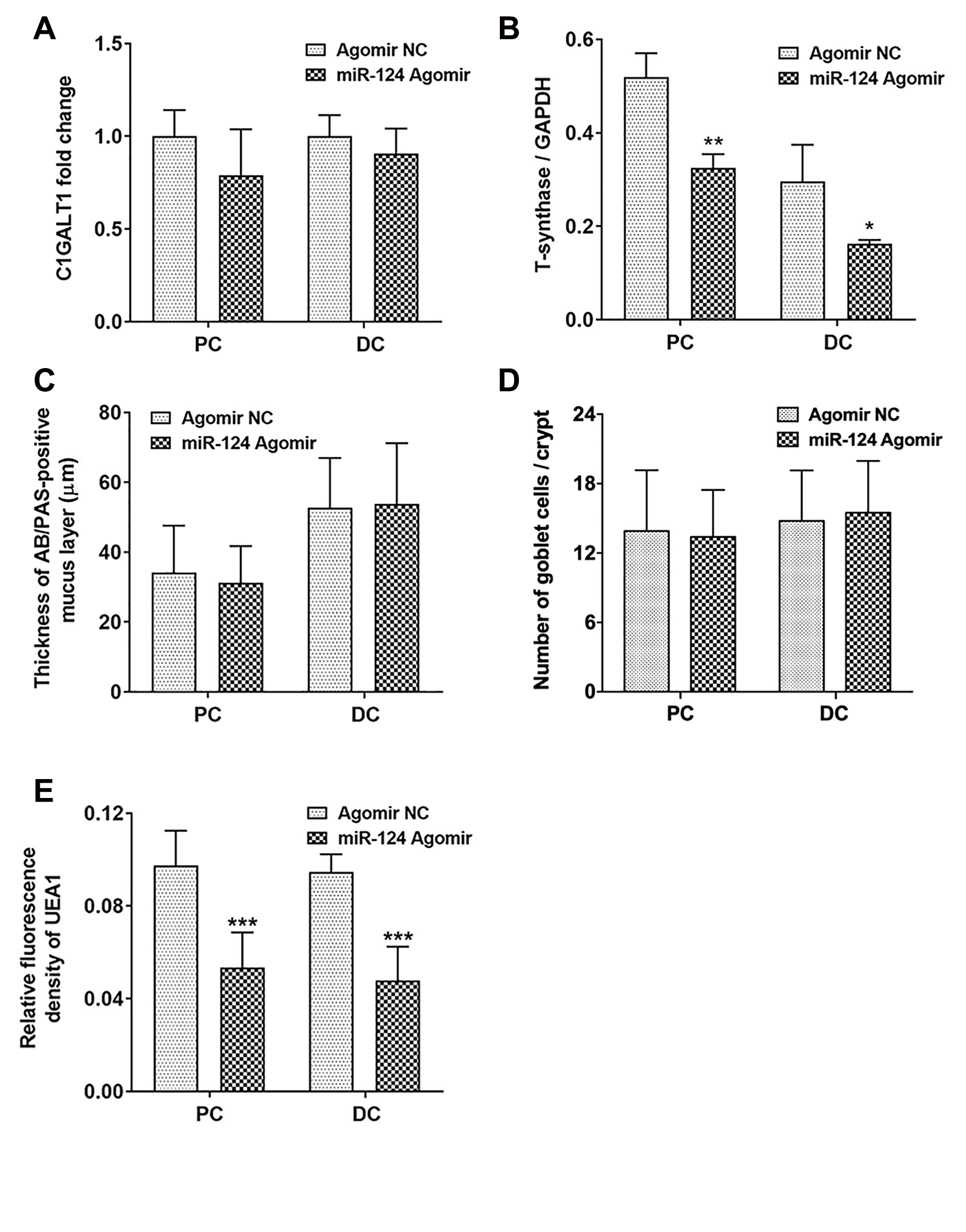

Supplement: Supplementary file 4 — Fig S4 [file ACEL-19-e13252-s004.tif]

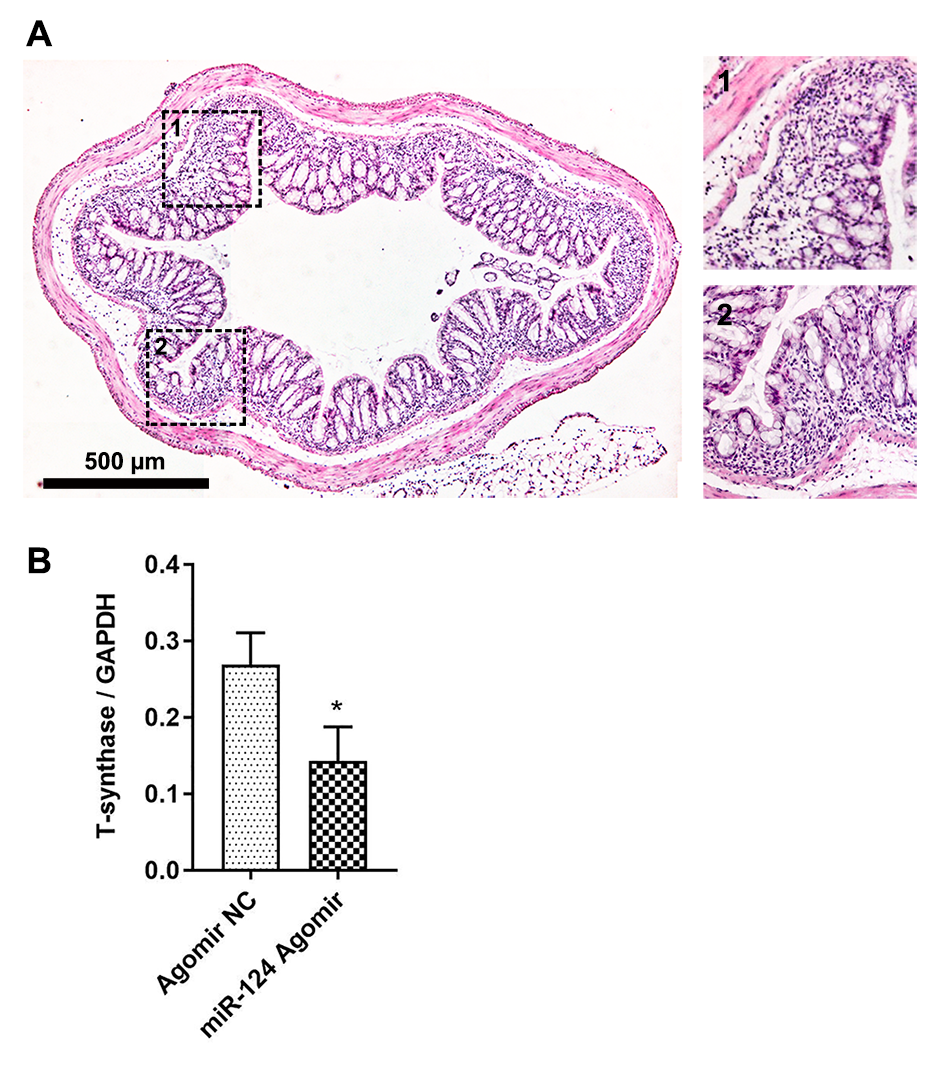

Supplement: Supplementary file 5 — Fig S5 [file ACEL-19-e13252-s005.tif]
