# Supplementary material for: Elevated miR‐124‐3p in the aging colon disrupts mucus barrier and increases susceptibility to colitis by targeting T‐synthase
Source: Aging Cell. 2020 Oct 11;19(11):e13252. doi: 10.1111/acel.13252 (PMC7681053; doi:10.1111/acel.13252)
Supplement: Supplementary file 6 — Table S1 [file ACEL-19-e13252-s006.doc]

**Supplemental table 1 Primer sequences used in the study**

| Gene name | Primers  (forward/reverse; F/R) | Sequence 5’-3’ |
| --- | --- | --- |
| Bacteria V3 region  (338F-518R) | F | ACTCCTACGGGAGGCAGCAGT |
|  | R | GTATTACCGCGGCTGCTGGCAC |
| E. coli | F | GTTAATACCTTTGCTCATTGA |
|  | R | ACCAGGGTATCTAATCCTGTT |
| H. pylori | F | CTGGAGAGACTAAGCCCTCC |
|  | R | ATTACTGACGCTGATTGTGC |
| H. hepaticus | F | GCATTTGAAACTGTTACTCTG |
|  | R | CTGTTTTCAAGCTCCCC |
| Lactobacillus | F | GAGGCAGCAGTAGGGAATCTTC |
|  | R | GGCCAGTTACTACCTCTATCCTTCTTC |
| Bifidobacterium | F | AGGGTTCGATTCTGCTCAG |
|  | R | CATCCGGCATTACCACCC |
| 18S | F | GTAACCCGTTGAACCCCATT |
|  | R | CCATCCAATCGGTAGTAGCG |
| Mouse C1GALT1 | F | TGCAGATTCCAGCCAACATAAAGATGA |
|  | R | AGCTTTGACATGTTTGGCCTTTTTCTC |
| Mouse C1GALT1C1 | F | CACCACTTAATCAAAGGTTATCTACCAA |
|  | R | AGGACCCTCTATGGGAGGATAATAG |
| Mouse TNF-α | F | GATTATGGCTCAGGGTCCAA |
|  | R | GCTCCAGTGAATTCGGAAAG |
| Mouse IL-1β | F | TGCCACCTTTTGACAGTGATG |
|  | R | TGATGTGCTGCTGCGAGATT |
| Mouse IL-6 | F | CCGGAGAGGAGACTTCACAG |
|  | R | CAGAATTGCCATTGCACAAC |
| Mouse GAPDH | F | TGCACCACCAACTGCTTAG |
|  | R | GGATGCAGGGATGATGTTC |
| Human C1GALT1 | F | CAAAATACGACCCTGAAGAACC |
|  | R | GCATCTCCCCAGTGCTAAGTC |
| Human C1GALT1C1 | F | TTTGAAGGGTGTGATGCTTG |
|  | R | ATGCGCTCATCCTCTGAAAT |
| Human TNF-α | F | TCCTTCAGACACCCTCAACC |
|  | R | AGGCCCCAGTTTGAATTCTT |
| Human IL-1β | F | GGGCCTCAAGGAAAAGAATC |
|  | R | TTCTGCTTGAGAGGTGCTGA |
| Human IL-6 | F | TACCCCCAGGAGAAGATTCC |
|  | R | TTTTCTGCCAGTGCCTCTTT |
| Human GAPDH | F | AGAAGGCTGGGGCTCATTTG |
|  | R | AGGGGCCATCCACAGTCTTC |
| miR-124-3p | F | CGCGTAAGGCACGCGGTG |
|  | R | ATCCAGTGCAGGGTCCGAGG |
| U6 | F | CTCGCTTCGGCAGCACA |
|  | R | AACGCTTCACGAATTTGCGT |
| miR-124-3p (stem-loop) | GTCGTATCCAGTGCAGGGTCCGAGGTATTCGCACTGGATACGACGGCATT | |
