# Supplementary material for: Elevated miR‐124‐3p in the aging colon disrupts mucus barrier and increases susceptibility to colitis by targeting T‐synthase
Source: Aging Cell. 2020 Oct 11;19(11):e13252. doi: 10.1111/acel.13252 (PMC7681053; doi:10.1111/acel.13252)
Supplement: Supplementary file 7 — Supplementary Material [file ACEL-19-e13252-s007.doc]

**Supplemental figure legends**

**Supplemental Figure 1 Aged colon exhibits thinner mucus layer.**

**A, B** The statistical analysis of thickness of AB/PAS- (A) and MUC2-positive (B) mucus layer in young and old mice. **C** Quantification of goblet cells per crypt. Data are presented as mean ± SD. *** *P* < 0.001 compared with 2-mo-old mouse group. Mann-Whitney test for (A, B, C). n=5 mice per group for morphometric analysis.

**Supplemental Figure 2 Aged distal colon shows loss of *O*-glycans and lower level of T-synthase.**

**A, B** Relative immunofluorescence density of UEA1 (A) and Tn (B) in the distal colonic mucosa of 2-, 16- and 24-mo-old mice. **C-E** Western blot densitometric analysis of Tn/sTn antigen (C), T-synthase (D) and Cosmc (E) protein expressions. Data are presented as mean ± SD. ** *P* < 0.01, *** *P* < 0.001 compared with 2-mo-old mouse group or young people group; ## *P* < 0.01, ### *P* < 0.001 compared with 16-mo-old mouse group. Student’s *t*-test for (C, D Ms PC and Hu, and E Ms PC and Hu), and ANOVA and LSD test for (A, B,D Ms DC, and E Ms DC). n=5 mice per group for morphometric analysis (A, B); n=8 mice or 7 human samples per group for molecular-biological analysis (C-E). Abbreviations: Ms, mouse; Hu, human; PC, proximal colon; DC, distal colon.

**Supplemental Figure 3 *MiR-124-3p* downregulates T-synthase protein expression.**

**A, B** GV306 luciferase reporter vector (A) and GV251-*miR-124-3p* construct (B) used in the dual-luciferase reporter assay. **C** Western blot densitometric analysis of T-synthase protein expression. Data are presented as mean ± SD. *** *P* < 0.001 compared with mimic NC group (ANOVA and LSD test). n=3 per group.

**Supplemental Figure 4 Mice overexpressing *miR-124-3p* show lower T-synthase protein expression.**

**A** Relative mRNA fold changes of *C1GALT1*. **B** Western blot densitometric analysis of T-synthase protein expression. **C** The statistical analysis of thickness of AB/PAS-positive mucus layer. **D** Quantification of goblet cells per crypt. **E** Relative immunofluorescence density of UEA1 in the colonic mucosa. Data are presented as mean ± SD. * *P* < 0.05, ** *P* < 0.01, *** *P* < 0.001 compared with agomir NC group. Student’s *t*-test for (A, B, E), and Mann-Whitney test for (C, D). n=6 mice per group for molecular-biological analysis (A, B); n=5 mice per group for morphometric analysis (C, D, E). Abbreviations: PC, proximal colon; DC, distal colon.

**Supplemental Figure 5 *MiR-124-3p* exacerbates DSS-induced colitis.**

**A** H&E images of the distal colonic mucosa from *miR-124-3p* agomir-treated mice showing the pathological structure and severe inflammation (high magnification 1, 2). **B** Western blot densitometric analysis of T-synthase protein expression. Data are presented as mean ± SD. * *P* < 0.05 compared with agomir NC group (Student’s *t*-test). n=5 mice per group.
