# Supplementary material for: Elevated miR‐124‐3p in the aging colon disrupts mucus barrier and increases susceptibility to colitis by targeting T‐synthase
Source: Aging Cell. 2020 Oct 11;19(11):e13252. doi: 10.1111/acel.13252 (PMC7681053; doi:10.1111/acel.13252)
Supplement: Supplementary file 8 — Supplementary Material [file ACEL-19-e13252-s008.doc]

**Supplementary experimental procedures**

**Assessment of disease severity in mice with DSS-induced colitis**

Mice were observed daily for morbidity and were scored daily to assess colitis activity by using the weight loss, stool consistency and bloody stool score. After sacrifice, mice were dissected, and the length of the large intestine was measured. Then, the colonic tissue was collected for subsequent experiments. Total scores were computed by summing individual scores across 3 categories of histological features associated with DSS-induced colonic mucosal pathology: degree of inflammatory cells infiltration, extent of injury and crypt damage (Table 1).

**H&E staining**

The tissues were fixed in 4% paraformaldehyde, dehydrated in ethanol, embedded in parafﬁn, and sectioned (5 μm) using a paraffin microtome (Leica RM 2135, Leica, Germany). The sections were stained with hematoxylin and eosin according to the standard procedure.

**AB/PAS staining**

Colons from mice without washing were fixed in methanol-Carnoy’s fixative (60% methanol, 30% chloroform, 10% acetic acid). Fixed tissues were embedded in paraffin and cut into 5-μm sections. Then the sections were dewaxed, hydrated and stained with AB/PAS. Strongly acidic mucins are stained blue, neutral mucins magenta and mixtures of both purple.

**Immunofluorescence staining**

Frozen sections (5 μm) were fixed in 4% paraformaldehyde for 20 min. Non-specific binding was blocked with 1% bovine serum albumin (BSA, Cat.No.V900933, Sigma-Aldrich, USA) for 1 h, followed by incubation with primary (overnight at 4 °C) and secondary antibodies (1 hours at 25 °C), as specified in Table 2. Negative-control sections were incubated in solutions lacking the primary antibody.

**Western blot analysis**

The total proteins were extracted from tissues using RIPA lysis buffer (Cat.No.C1053, Applygen, China) containing protease inhibitor cocktail (Cat.No.P8340, Sigma-Aldrich, USA) and phosphatase inhibitor cocktail (Cat.No.P5726, Sigma-Aldrich, USA). The protein concentration was quantified using a BCA protein assay kit (Cat.No.P1511, Applygen, China). SDS-PAGE was performed using a 10% separating acrylamide gel and a 4% stacking acrylamide gel. Equal amounts (20-40 μg) of total protein from each sample were electrophoresed on SDS-PAGE and transferred to a nitrocellulose membrane. After blocking with Tris-buffered saline containing 0.05% Tween-20 (TBST) and 5% non-fat dry milk or 5% BSA for 1 h, the membranes were incubated with primary and secondary antibodies as specified in Table 2. The protein bands were detected using enhanced chemiluminescence (Cat.No.1705060, Bio-Rad Laboratories, USA) and viewed in Fusion FX Vilber Lourmat (France). GAPDH and α-Tubulin were used as internal controls.

**RNA and genomic DNA extraction and real-time PCR**

The total RNA was extracted from flash-frozen tissues or cell lines using TRIzol reagent (Cat.No.15596026, Life Technologies, USA) and converted to cDNA in a Veriti 96-well Thermal Cycler (Applied Biosystems, USA) using a TaqMan™ MicroRNA Reverse Transcription Kit (Cat.No.4366596, Applied Biosystems) or All-In-One RT MasterMix (Cat.No.G486, ABM, Canada). For bacterial 16S rRNA analysis, bacterial genomic DNA was extracted from flash-frozen tissues using a DNeasy Blood and Tissue Kit (Cat.No.69504, Qiagen, Germany). Real-time qPCR was performed on an ABI 7500 real-time PCR system (Life Technologies, USA) using Maxima SYBR Green/ROX qPCR Master Mix (Cat.No.K0222, Thermo Scientific, USA). The primers used are listed in Table S1. Twenty-five microliter reactions were incubated at 95 °C for 10 min, followed by 40 cycles at 95 °C for 10 s, 60 °C for 10 s, and 72 °C for 40 s. The relative fold change of the genes was calculated using the 2^−ΔΔCt^ method. All expression data were normalized relative to U6, GAPDH, or 18S rRNA, which were used as internal controls.
